# Soluble ADAM33 initiates airway remodeling to promote susceptibility for allergic asthma in early life

**DOI:** 10.1172/jci.insight.87632

**Published:** 2016-07-21

**Authors:** Elizabeth R. Davies, Joanne F.C. Kelly, Peter H. Howarth, David I. Wilson, Stephen T. Holgate, Donna E. Davies, Jeffrey A. Whitsett, Hans Michael Haitchi

**Affiliations:** 1 The Brooke Laboratory, Academic Unit of Clinical and Experimental Sciences, Faculty of Medicine, University of Southampton, Southampton, United Kingdom.; 2 Division of Pulmonary Biology, Cincinnati Children’s Hospital Medical Center, Cincinnati, Ohio, USA.; 3 National Institute for Health Research (NIHR) Southampton Respiratory Biomedical Research Unit, University Hospital Southampton NHS Foundation Trust, Southampton, United Kingdom.; 4 Institute for Life Sciences and; 5 Centre for Human Development, Stem Cells and Regeneration, Human Genetics, Faculty of Medicine, University of Southampton, Southampton, United Kingdom.

## Abstract

Asthma is a chronic inflammatory airways disease that usually begins in early life and involves gene-environment interactions. Although most asthma exhibits allergic inflammation, many allergic individuals do not have asthma. Here, we report how the asthma gene a disintegrin and metalloprotease 33 (*ADAM33*) acts as local tissue susceptibility gene that promotes allergic asthma. We show that enzymatically active soluble ADAM33 (sADAM33) is increased in asthmatic airways and plays a role in airway remodeling, independent of inflammation. Furthermore, remodeling and inflammation are both suppressed in *Adam33*-null mice after allergen challenge. When induced in utero or added ex vivo, sADAM33 causes structural remodeling of the airways, which enhances postnatal airway eosinophilia and bronchial hyperresponsiveness following subthreshold challenge with an aeroallergen. This substantial gene-environment interaction helps to explain the end-organ expression of allergic asthma in genetically susceptible individuals. Finally, we show that sADAM33-induced airway remodeling is reversible, highlighting the therapeutic potential of targeting ADAM33 in asthma.

## Abstract 2

Loss of ADAM33 suppresses airway remodeling and allergic inflammation in mice, suggesting the therapeutic potential of targeting ADAM33 in asthma.

## Introduction

Even though our insight into the pathophysiology of asthma has increased, it still remains a substantial health and economic burden, with 300 million patients suffering from asthma worldwide. It is a complex disease involving gene-environment interactions, and most individuals with asthma exhibit allergen-induced Th2-type inflammatory responses associated with increased expression of cytokines from the IL-4 gene cluster linked to atopy (1). Asthma is also characterized by structural remodeling and thickening of the inner airway wall, involving an increase in bronchial smooth muscle, collagen deposition, and neoangiogenesis. The classical paradigm of allergic asthma (2) suggests that Th2-type–driven airway inflammation plays a causal role in airway remodeling (i.e., inflammation precedes remodeling) (3) to result in the typical asthmatic phenotype (4–6). However, the early use of inhaled corticosteroids for wheezing in preschool children does not affect the natural history of asthma or wheeze later in childhood (7). Similarly, although treatment of mild-to-moderate asthmatic children with antiinflammatory drugs provides asthma control and reduces airway responsiveness to methacholine, this fails to improve lung function when compared with placebo (8). Since airway remodeling (9) and bronchial smooth muscle (10) are increased in the airways of young children who subsequently progress to develop asthma, these studies suggest distinct mechanisms that affect remodeling early in the disease course, but the causal factors are unknown (11).

While the genetic susceptibility to atopy alone cannot explain asthma (12), the functional contribution of other asthma susceptibility genes to disease pathogenesis is poorly understood (13). A disintegrin and metalloprotease 33 (*ADAM33*) is a susceptibility gene associated with asthma and bronchial hyperresponsiveness (BHR) (14, 15), a finding that has been replicated in multiple populations (16). *ADAM33* alleles are also associated with an increased risk for COPD (17), accelerated lung function decline across the life course (18, 19), and impaired lung function early in life (20), suggesting a role in airway remodeling. ADAM33 is mainly expressed in airway smooth muscle, fibroblasts, and mesenchymal progenitor cells (21, 22), with each playing a role in asthma-related airway remodeling (23, 24). Although it is a membrane-anchored protein, ADAM33 can be detected as a soluble protein (sADAM33) in asthmatic airways in which high levels of sADAM33 are correlated with reduced lung function (25). Little is understood about the contribution of sADAM33 to the pathogenesis of asthma, although it is known that sADAM33 protein is induced in utero by maternal allergy (26) and the enzymatically active recombinant protein is proangiogenic (27). Herein, we provide insight into how the disease-related sADAM33 protein promotes airway remodeling without inflammation; we also demonstrate a substantial gene-environment interaction in which sADAM33-induced airway remodeling in early life affects the susceptibility of the airway tissue to environmental allergens to promote allergic airway inflammation and BHR. Finally, we show that ADAM33-driven airway remodeling is reversible, highlighting the potential for sADAM33 as a target for disease-modifying therapy.

## Results

### sADAM33 is increased in asthma.

**Figure 1:**
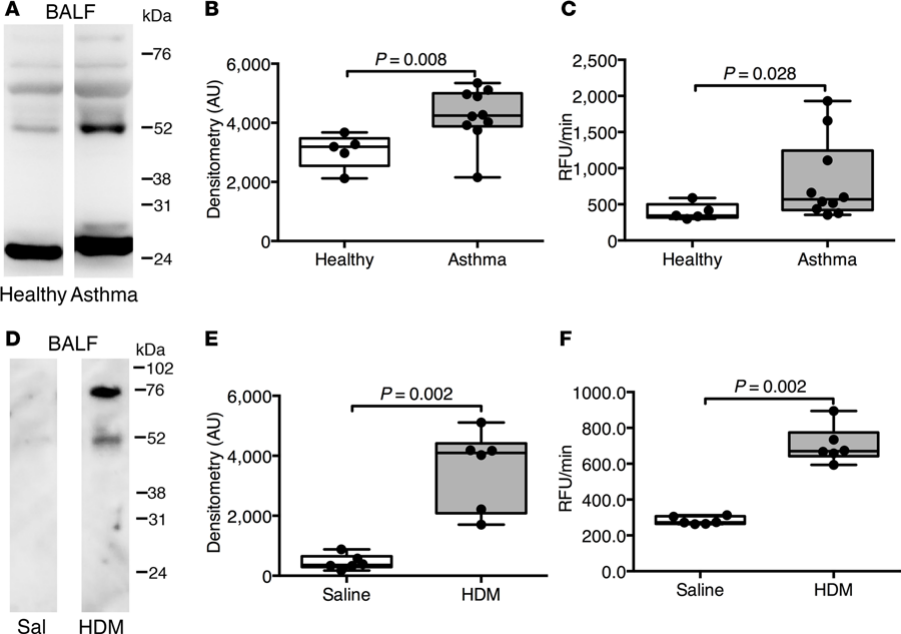
Increased soluble ADAM33 (sADAM33) enzymatic activity in bronchoalveolar lavage fluid (BALF) in human asthma and allergic mice. (**A**) Western blotting of BALF proteins from healthy (*n* = 5) and asthmatic (*n* = 10) subjects using an antibody recognizing the metalloprotease domain of human ADAM33; representative blots are shown. (**B**) Combined ADAM33-immunoreactive bands (at approximately 25 kDa and between approximately 52 and 76 kDa) were analyzed by densitometry in arbitrary units (AU) (Mann Whitney test). (**C**) Fluorescence resonance energy transfer (FRET) peptide cleavage assay for ADAM33-specific enzymatic activity in BALF from healthy (*n* = 5) and asthmatic donors (*n* = 10), expressed as relative fluorescence units per minute (RFU/min) (Mann Whitney test). (**D**) Immunoblotting of BALF protein from WT mice challenged with house dust mite (HDM) extract or saline (Sal) (*n* = 6 per group) with an antibody against mouse ADAM33 extracellular domain. (**E**) Semiquantitative analysis (bands at approximately 52 and 76 kDa) by densitometry (Mann Whitney test). (**F**) FRET peptide cleavage assay using murine BALF from HDM-challenged mice or saline controls (*n* = 6 per group) (Mann Whitney test). Box plots show medians and 25th to 75th percentiles, and whiskers represent minimum and maximum values; all data points are shown. Results are from 3 independent experiments (**D**–**F**). Full unedited Western blots are available in the Supplemental Material.

Since ADAM33 contains a metalloprotease (MP) domain, we tested if sADAM33 in bronchoalveolar lavage fluid (BALF) is enzymatically active. Consistent with a previous report (25), immunoreactive sADAM33 was detected in BALF using an antibody against the MP domain, with a strong band at approximately 25 kDa (MP domain), another at approximately 52 kDa (unprocessed MP domain, i.e., Pro-MP), and other minor bands of higher molecular weight (ectodomain fragments containing the MP domain) (Figure 1A). The 52-kDa band was confirmed as the unprocessed Pro-MP domain using an antibody against the Pro domain (Supplemental Figure 1A; supplemental material available online with this article; doi:10.1172/jci.insight.87632DS1). The bands were similar to those previously characterized in BALF from sarcoid patients (28) and were significantly increased in asthma (Figure 1B and Supplemental Figure 1B). Neither ADAM33 antibody cross-reacted with recombinant ADAM8 and ADAM12 (Supplemental Figure 2, A–C), which have been associated with asthma (29–31) and show high homology with ADAM33. Furthermore, using an ADAM33-specific fluorescence resonance energy transfer (FRET) peptide cleavage assay, ADAM33 enzymatic activity was increased in asthma (Figure 1C). The presence of active sADAM33 in BALF was independent of corticosteroid treatment or the airway inflammatory cell profile (Supplemental Table 1). We also analyzed BALF from inbred mice after sensitization and challenge with house dust mite (HDM) extract allergen (Supplemental Figure 3), which induces features of asthma in murine lungs. Western blotting using an antibody to murine ADAM33 antibody (26) revealed distinct protein bands similar to the human ADAM33 at approximately 52 to 76 kDa (Figure 1, D and E), smaller than the processed form of full-length mouse ADAM33 (approximately 110 kDa) and consistent with its proteolytic cleavage from its membrane-bound form to release sADAM33 ectodomain into the airways (32). Similar to the findings in human BALF, enzymatic activity of murine sADAM33 was significantly increased in the BALF of lungs from HDM-challenged mice when compared with the equivalent control mice (Figure 1F).

### sADAM33 causes airway remodeling.

**Figure 2:**
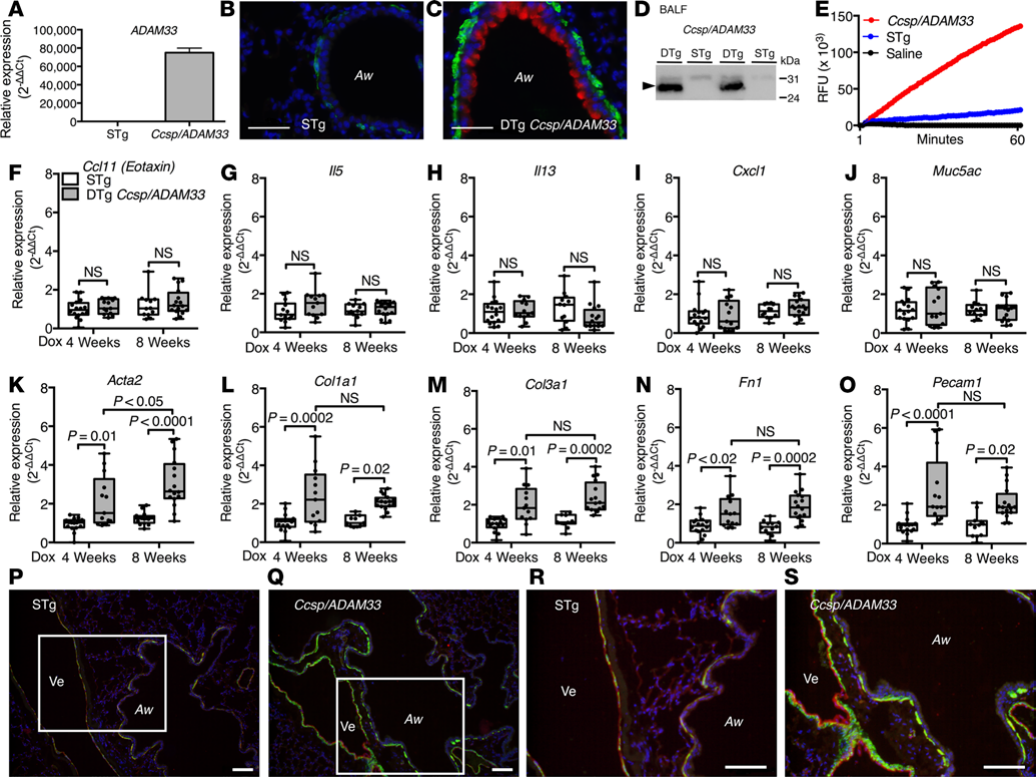
Transgenic expression of human soluble ADAM33 (sADAM33) causes airway remodeling. (**A**) Human *ADAM33* mRNA in single-transgenic (STg) littermate control and double-transgenic (DTg) *Ccsp/ADAM33* mouse lungs. (**B** and **C**) Representative immunofluorescence staining images for human ADAM33 (red), ACTA2/αSMA (green), and nuclei (blue) in lungs from (**B**) STg littermate control and (**C**) DTg *Ccsp/ADAM33* mice. Scale bar: 50 μm. (**D**) Western blotting for human ADAM33 in BALF from DTg *Ccsp/ADAM33* or STg control mice. (**E**) Fluorescence resonance energy transfer (FRET) peptide cleavage assay for ADAM33 enzymatic activity in bronchoalveolar lavage fluid (BALF) from DTg *Ccsp/ADAM33* (red) mice, STg (blue) mice, or saline controls (black). Representative traces are shown. (**F–O**) Relative mRNA expression in whole-lung lobe lysates from adult DTg *Ccsp/ADAM33* mice (gray bars) after induction of human ADAM33 for 4 (*n* = 13) or 8 weeks (*n* = 16) versus STg littermate controls (white bars) (*n* = 16 or *n* = 12, respectively): (**F**) *Ccl11*, (**G**) *Il5*, (**H**) *Il13*, (**I**) *Cxcl1*, (**J**) *Muc5ac,* (**K**) *Acta2*, (**L**) *Col1a1*, (**M**) *Col3a1*, (**N**) *Fn1*, and (**O**) *Pecam1* (2-way ANOVA, Tukey’s multiple comparison test). Box plots show medians and 25th to 75th percentiles, and whiskers represent minimum and maximum values; all data points are shown. Results are from 3 independent experiments (**F–O**). Representative immunofluorescence staining for ACTA2/αSMA (green), PECAM1 (red), and nuclei (blue) in lungs from (**P** and **R**) STg littermate control or (**Q** and **S**) DTg *Ccsp/ADAM33* mice after 8 weeks of transgene expression. White rectangles in **P** and **Q** are shown at higher magnification in **R** and **S**. Aw, airway; Ve, vessel. Scale bar: 100 μm. Results are representative of 3 independent experiments (**P–S**). Full unedited Western blots are available in the Supplemental Material.

To assess directly the function of sADAM33 in vivo, a doxycycline-inducible (Dox-inducible) human sADAM33 transgenic mouse model was generated by injecting a linearized *TRES-human-ADAM33-SS-PRO-MP-3Flag* construct (Supplemental Figure 4, A–H) into FVB/N mouse pronuclei. By crossing the founder mice with *Ccsp-rtTA*(line 2) (33) mice, we obtained expression of human s*ADAM33* mRNA (Figure 2A) in the lungs of Dox-fed double-transgenic (DTg) (*Ccsp/ADAM33*) mice but not in single-transgenic (STg) littermate controls. Epithelial expression of human sADAM33 protein in Dox-induced DTg *Ccsp/ADAM33* mice was confirmed by immunofluorescence staining (Figure 2, B and C), and enzymatically active sADAM33 was demonstrated in BALF (Figure 2, D and E).

When 6- to 8-week-old mice were fed a diet containing Dox for 4 or 8 weeks to induce transgene expression of human sADAM33 (Supplemental Figure 5A), there were no significant changes in the airway expression of inflammatory (*Ccl11* [also known as *Eotaxin*], *Il5*, *Il13*, *Cxcl1* [also known as *Kc*]) or goblet cell (*Muc5ac*) markers (Figure 2, F–J), and there was no evidence of inflammatory cells in the BALF of these mice (Supplemental Figure 5B). In contrast, α-smooth muscle actin (*Acta2;* also known as *α-Sma*) mRNA and that of other genes linked to airway remodeling, including collagen 1 and 3 (*Col1a1* and *Col3a1*), fibronectin (*Fn1*), and platelet/endothelial cell adhesion molecule 1 (*Pecam1*; also known as *CD31*), were increased in whole lungs of DTg *Ccsp/ADAM33* mice compared with STg littermate controls (Figure 2, K–O). Immunofluorescence staining for ACTA2 and PECAM1 in DTg *Ccsp/ADAM33* mice revealed increased smooth muscle surrounding the airways and airway vessels (Figure 2, Q and S) compared with STg littermate controls (Figure 2, P and R). Together, these data support promyogenic and proangiogenic functions (27) for sADAM33. However, these remodeling changes failed to elicit BHR in response to inhaled methacholine (Supplemental Figure 5C).

### sADAM33 causes airway “remodeling” in developing lung.

**Figure 3:**
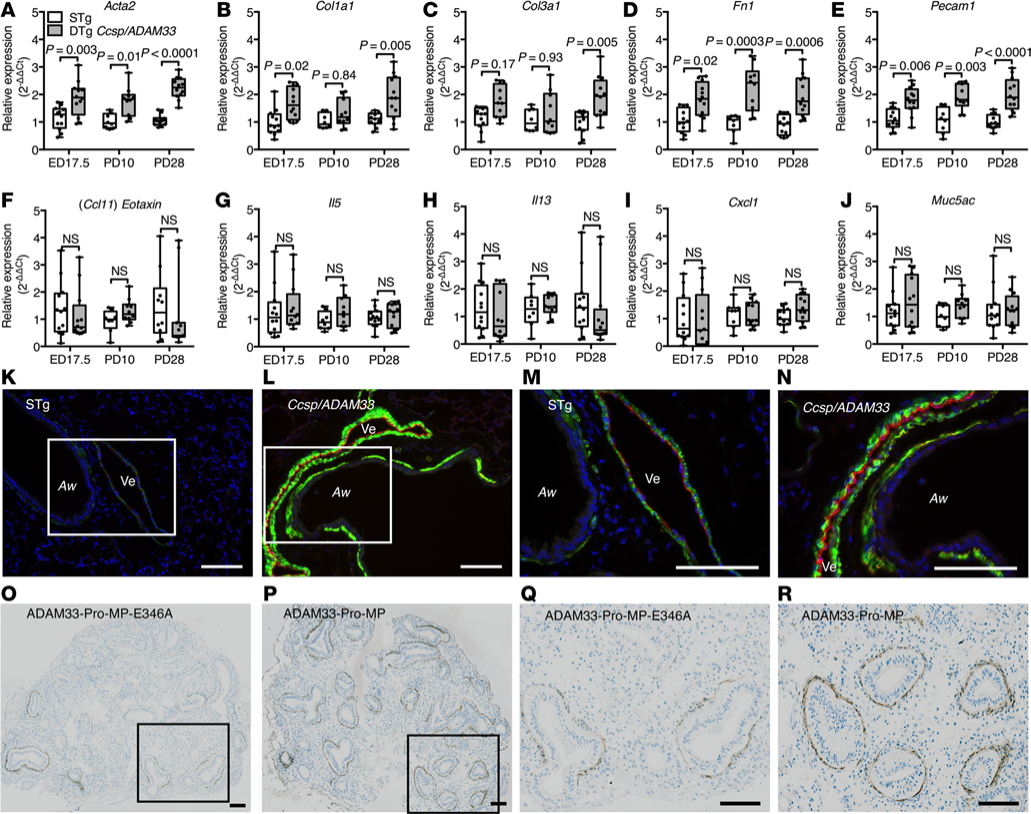
Human soluble ADAM33 (sADAM33) causes airway “remodeling” in developing lungs. (**A–J**) Reverse-transcription quantitative PCR (RT-qPCR) for remodeling and inflammatory gene expression in whole-lung lobe lysates from double-transgenic (DTg) *Ccsp/ADAM33* or single-transgenic (STg) littermate control mice at embryonic day 17.5 (ED17.5) (*n* = 12/12) and 10 days postpartum (PD10) (*n* = 8/10) and 28 days postpartum (PD28) (*n* = 12/12) in which transgene expression was induced by feeding mice doxycycline during pregnancy and up to 4 weeks after birth: (**A**) *Acta2*, (**B**) *Col1a1*, (**C**) *Col3a1*, (**D**) *Fn1* (**E**) *Pecam1* (**F**) *Ccl11*, (**G**) *Il5*, (**H**) *Il13*, (**I**) *Cxcl1*, and (**J**) *Muc5ac* (2-way ANOVA, Tukey’s multiple comparison test). Box plots show medians and 25th to 75th percentiles, and whiskers represent minimum and maximum values; all data points are shown. Representative immunofluorescence staining for ACTA2/αSMA (green), PECAM1 (red), and nuclei (blue) in lungs from (**K** and **M**) STg littermate control or (**L** and **N**) DTg *Ccsp/Adam33* mice after ADAM33 transgene expression for 4 weeks postpartum. White rectangles in **K** and **L** are shown at higher magnification in **M** and **N**. Aw, airway; Ve, vessel. Representative immunohistochemistry staining for ACTA2/αSMA (brown) in sections of human embryonic lung explants cultures from 8 to 10 weeks after conception (*n* = 3) in the presence of (**O** and **Q**) recombinant inactive mutant (E346A) ADAM33-Pro-metalloprotease (ADAM33-Pro-MP) and (**P** and **R**) enzymatically active ADAM33-Pro-MP. Black rectangles in **O** and **P** are shown at higher magnification in **Q** and **R**. Scale bar: 100 μm. Results are representative of 3 independent experiments.

Since sADAM33 protein can be induced in utero by maternal allergy (26), we also determined the effect of inducing human sADAM33 during lung development by feeding transgenic mice a Dox diet during pregnancy and for up to 4 weeks after birth (Supplemental Figure 5D). This caused sADAM33 to be expressed in the fetal lungs from around embryonic day (ED) 14/15 (corresponding to the time when the CCSP promoter becomes active; ref. 34) until the mice were euthanized for analysis. From as early as ED17.5, the airway remodeling genes *Acta2*, *Col1a1*, *Col3a1*, *Fn1*, and *Pecam1* were all increased in whole lungs of DTg *Ccsp/ADAM33* mice compared with STg littermate controls (Figure 3, A–E), while the inflammatory markers, *Ccl11*, *Il5*, *Il13*, and *Cxcl1*, and goblet cell marker, *Muc5ac*, were not affected (Figure 3, F–J), as was the case in the adult transgenic mice (Figure 2, F–O). Immunofluorescence histochemistry in lungs of 4-week-old *Ccsp/ADAM33* mice confirmed marked induction of airway remodeling involving increased airway and vascular smooth muscle compared with control mice (Figure 3, K–N). As observed with the adult transgenic mice, these remodeling changes failed to elicit inflammation (Supplemental Figure 5E) or BHR in response to inhaled methacholine (Supplemental Figure 5F).

Importantly, we also found that exposure of human embryonic lung explants to highly purified enzymatically active recombinant sADAM33-Pro-MP in vitro caused increased bronchial smooth muscle in developing airways; this effect was dependent on the catalytic activity of the enzyme (Figure 3, P and R), since mutation of the active site (E346A) was inactive and failed to reproduce this effect (Figure 3, O and Q).

### Allergen-induced asthma-like changes are inhibited in Adam33^–/–^ mice.

**Figure 4:**
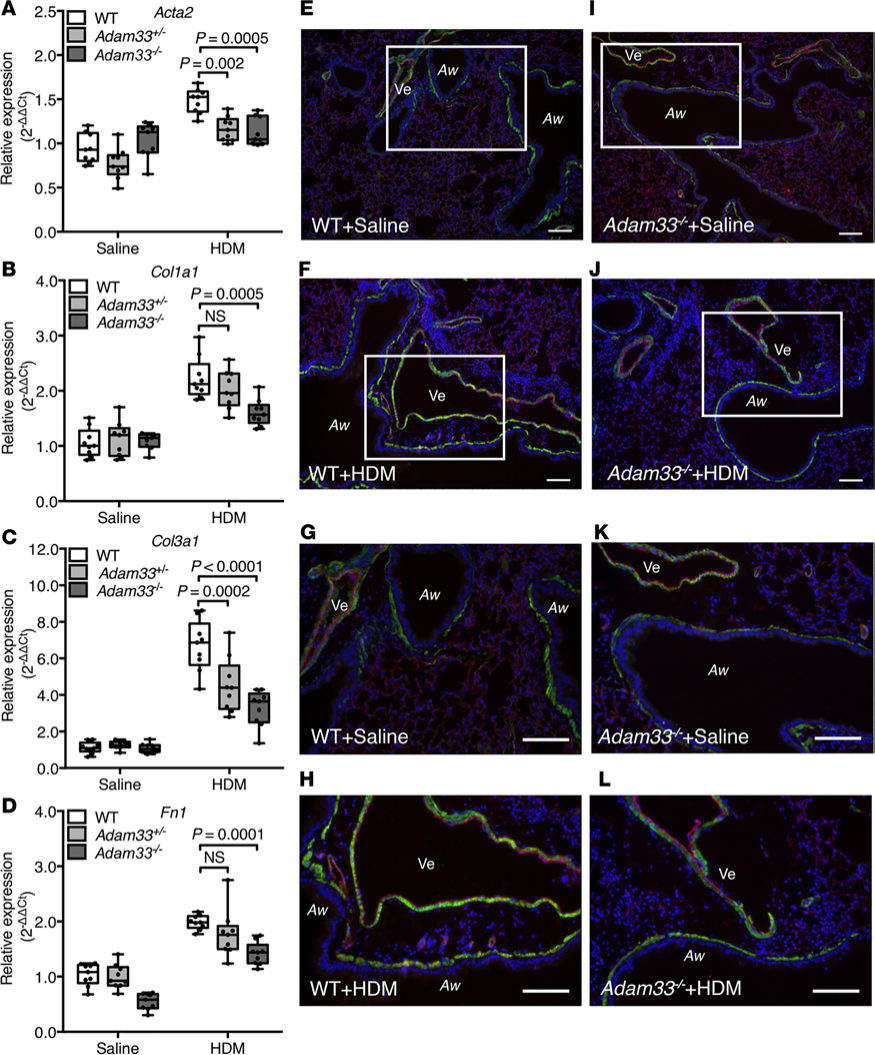
Suppression of house dust mite (HDM) extract-induced airway remodeling in *Adam33^–/–^* mice. (**A–D**) Reverse-transcription quantitative PCR (RT-qPCR) using whole-lung lysates from WT, heterozygote (*Adam33*^+/-^), and *Adam33*^–/–^ mice challenged with saline or HDM extract: (**A**) *Acta2*, (**B**) *Col1a1*, (**C**) *Col3a1*, and (**D**) *Fn1* (*n* = 9 per group; 2-way ANOVA Tukey’s multiple comparison test). (**E–L**) Representative immunofluorescence staining for ACTA2/αSMA (green), PECAM1 (red), and nuclei (blue) in tissue sections from mouse lungs after in vivo challenge with saline or HDM extract: (**E** and **G**) WT+saline, (**F** and **H**) WT+HDM, (**I** and **K**) *Adam33*^–/–^+saline, and (**J** and **L**) *Adam33*^–/–^+HDM. White rectangles in **E**, **F**, **I**, and **J** are shown in **G**, **H**, **K**, and **L** at higher magnification. Aw, airway; Ve, vessel. Scale bar: 100 μm. Results are representative of 3 independent experiments (**E–L**).

**Figure 5:**
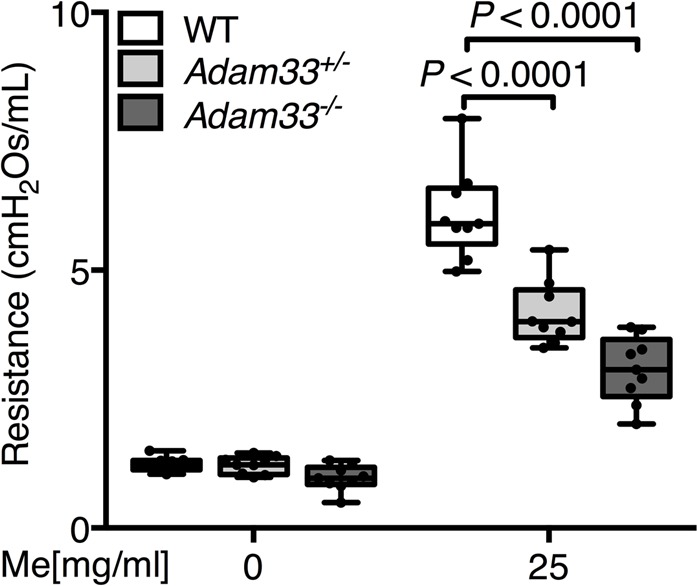
Suppression of house dust mite (HDM) extract-induced airway hyperresponsiveness in *Adam33^–/–^* mice. Airway resistance in response to methacholine (Me) in WT (white), *Adam33*^+/-^ (light gray), and *Adam33*^–/–^ (dark gray) mice following HDM exposure (*n* = 9 per group; 2-way ANOVA, Tukey’s multiple comparison test). Results are representative of 3 independent experiments.

**Figure 6:**
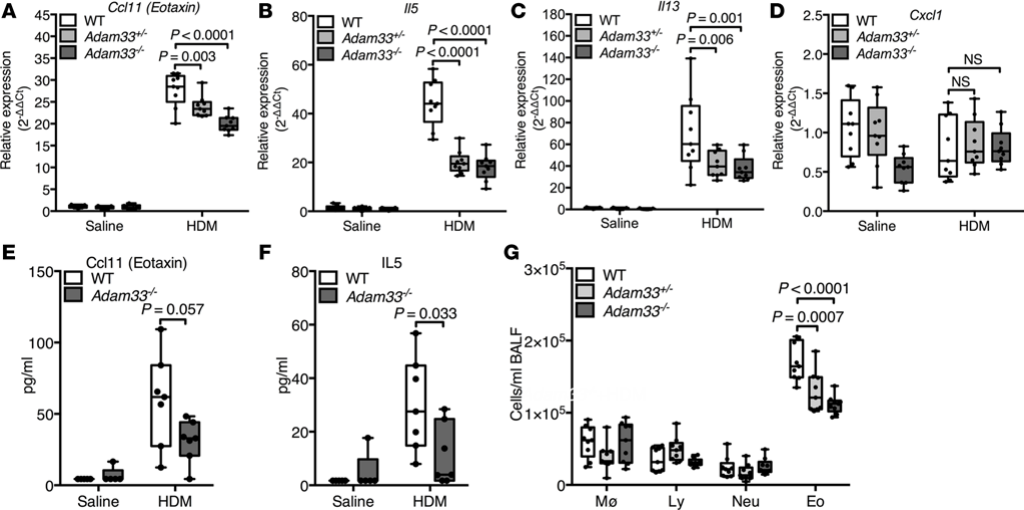
Suppression of house dust mite (HDM) extract-induced airway inflammation in *Adam33^–/–^* mice. (**A–D**) Reverse-transcription quantitative PCR (RT-qPCR) using whole-lung lysates from WT (white), heterozygote (*Adam33*^+/-^) (light gray), and *Adam33*^–/–^ (dark gray) mice challenged with saline or HDM extract: (**A**) *Ccl11*, (**B**) *Il5*, (**C**) *Il13*, and (**D**) *Cxcl1*; (*n* = 9 per group; 2-way ANOVA Tukey’s multiple comparison test). (**E** and **F**) Multiplex assay for CCL11 and IL-5 protein levels in bronchoalveolar lavage fluid (BALF) (*n* = 5 or 7 per group; 1-way ANOVA, Tukey’s multi comparison test). (**G**) Differential inflammatory cell counts for macrophages (MØ), lymphocytes (Ly), neutrophils (Neu), and eosinophils (Eo) in BALF from WT, *Adam33*^+/-^, and *Adam33*^–/–^ mice challenged with HDM (*n* = 9 per group; 2-way ANOVA Tukey’s multiple comparison test). Box plots show medians and 25th to 75th percentiles, and whiskers minimum to maximum; all data points are shown. Results are from 3 independent experiments.

To determine whether ADAM33 is required for allergen-induced airway remodeling, we evaluated *Adam33-*null (*Adam33^–/–^)* mice (Supplemental Figure 6) after intraperitoneal sensitization and intratracheal challenge with HDM allergen (Supplemental Figure 3). Although a previous report had failed to observe changes in ovalbumin-induced airway responses in *Adam33*^–/–^ mice (35), we investigated effects of HDM extract as a common human aeroallergen to model more closely human asthma. In WT mice, HDM challenge caused an increase in sADAM33 enzyme activity in BALF, whereas there was no significant effect in *Adam33^–/–^* mice (Supplemental Figure 7A), confirming the specificity of the ADAM33 FRET assay. Similarly, after HDM challenge, *Acta2*, *Col1a1*, *Col3a1*, and *Fn1* mRNAs were significantly increased in whole lungs of WT mice (Figure 4, A–D), but these mRNAs were suppressed in the *Adam33-*deleted mice in a gene dosage-dependent fashion. Compared with saline-treated control mice (Figure 4, E, G, I, and K), the HDM-induced increase in smooth muscle around the airways and vessels of WT mice (Figure 4, F and H) was suppressed in the lungs of HDM-challenged *Adam33*^–/–^ mice (Figure 4, J and L) and paralleled by reduced methacholine-induced BHR (Figure 5 and Supplemental Figure 7B). Of note, mRNAs encoding the Th2-type inflammatory genes, *Ccl11*, *Il5*, and *Il13* but not *Cxcl1,* were also suppressed in the HDM-challenged *Adam33*^–/–^ mice (Figure 6, A–D) and were associated with a decrease in CCL11 and IL-5 (Figure 6, E and F) and eosinophils (Figure 6G) in BALF. These data suggest a previously unappreciated role for sADAM33 in regulating Th2-type inflammation.

### Early-life induction of sADAM33 enhances allergen-induced asthma-like changes.

**Figure 7:**
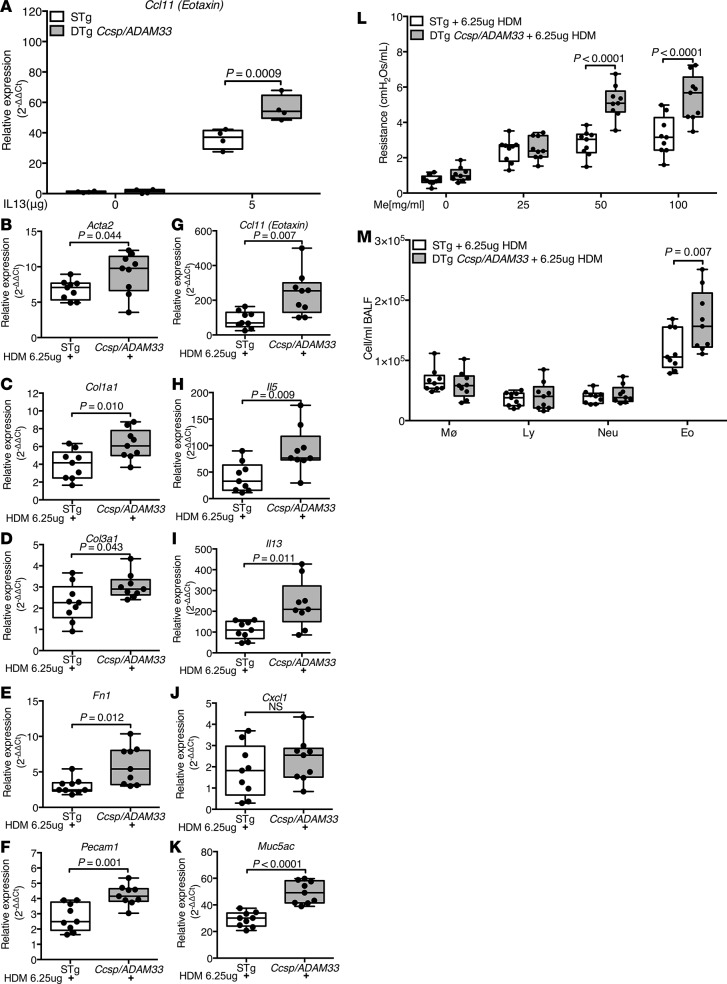
Soluble ADAM33 (sADAM33) augments airway responses to allergens. (**A**) Reverse-transcription quantitative PCR (RT-qPCR) for *Ccl11* mRNA expression in lung lobe lysates 24 hours after intratracheal installation of 5.0 μg murine IL-13 in double-transgenic (DTg) *Ccsp/ADAM33* (gray bars) and single-transgenic (STg) littermate control mice (white bars) after transgene induction for 4 weeks (*n* = 4/group; 2-way ANOVA, Tukey’s multiple comparison test). Results are from 1 experiment. (**B–M**) After transgene induction for 6 weeks, DTg *Ccsp/ADAM33* or STg control mice were sensitized and challenged with house dust mite (HDM) extract (6.5 μg) for analysis of gene expression, bronchial hyperresponsiveness (BHR), and inflammation. (**B–K**) RT-qPCR for relative mRNA expression (compared with saline-challenged mice) in lung lobe lysates from DTg *Ccsp/ADAM33* or STg control mice: (**B**) *Acta2*, (**C**) *Col1a1*, (**D**) *Col3a1*, (**E**) *Fn1*, (**F**) *Pecam1*, (**G**) *Ccl11*, (**H**) *Il5*, (**I**) *Il13*, (**J**) *Cxcl1*, and (**K**) *Muc5ac* (all *n* = 9 per group; unpaired Student’s *t* test or Mann Whitney test). (**L**) Airway resistance in response to increasing concentrations of methacholine (Me) and (**M**) differential inflammatory cell counts for macrophages (MØ), lymphocytes (Ly), neutrophils (Neu), and eosinophils (Eo) in bronchoalveolar lavage fluid (BALF) after HDM or saline challenge (*n* = 9 per group; 2-way ANOVA, Tukey’s multiple comparison test). Box plots show medians and 25th to 75th percentiles, and whiskers represent minimum and maximum values; all data points are shown. Results are from 3 independent experiments.

To test the hypothesis that sADAM33 enhances asthma-like responses in the presence of a Th2 stimulus, we treated *Ccsp/ADAM33* and STg littermate control mice with recombinant IL-13 (Supplemental Figure 8A) and found a significant increase in mRNA expression of the fibroblast-derived chemokine, *Ccl11*, in the sADAM33-expressing mice (Figure 7A). This amplification effect led us to consider the potential for early-life interactions between sADAM33 and Th2-type inflammation in response to low concentrations of allergen. We first performed a concentration-response experiment with HDM extract with a standard sensitization protocol (Supplemental Figure 3) to determine the amount of HDM extract (6.25 μg), which elicited minimal BHR, eosinophilia, and *Ccl11* and *Muc5ac* mRNA (Supplemental Figure 8, B–E) in WT mice. This low-dose allergen challenge protocol was then applied to DTg *Ccsp/ADAM33* and STg littermate control mice in which transgene expression was induced during lung development and early life (Supplemental Figure 9A). In response to HDM challenge, expression of genes mediating airway remodeling, *Acta2*, *Col1a1*, *Col3a1*, *Fn1*, and *Pecam1* (Figure 7, B–F); Th2 inflammatory cytokines, *Ccl11*, *Il5*, and *Il13* (Figure 7, G–I); and mucus production *Muc5ac* (Figure 7K), but not *Cxcl1* (Figure 7J), were increased in DTg *Ccsp/ADAM33* mice compared with equivalently treated STg littermate controls. Enhanced expression of inflammation and remodeling genes in the *Ccsp/ADAM33* mice was accompanied by increased airway resistance, as measured by BHR in response to methacholine (Figure 7L and Supplemental Figure 9, B and C), and accompanied by BALF eosinophilia (Figure 7M).

### Airway remodeling induced by sADAM33 is reversible.

**Figure 8:**
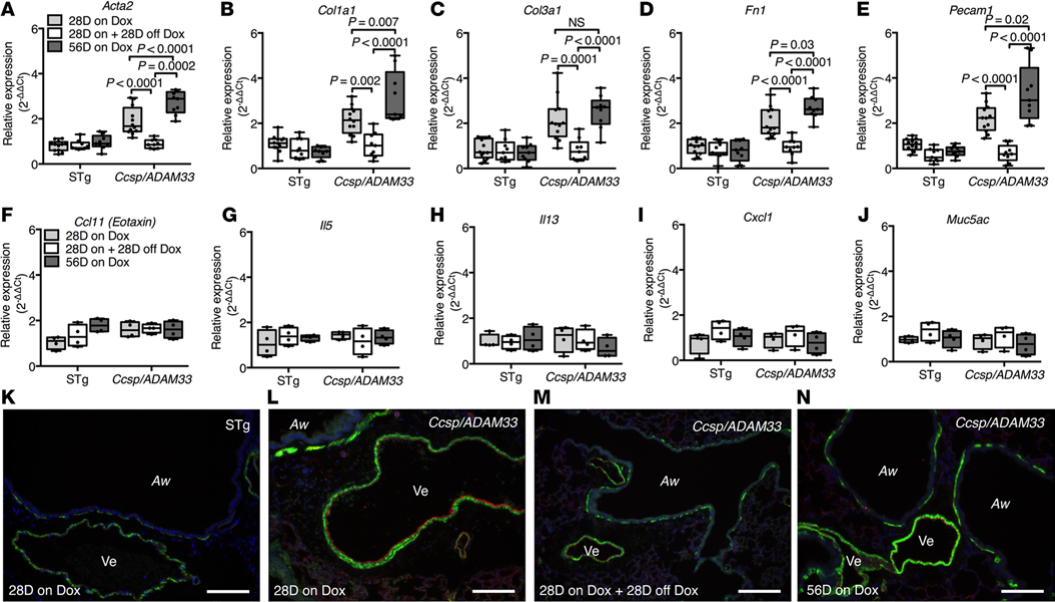
Airway remodeling induced by soluble ADAM33 (sADAM33) is reversible. (**A–J**) Reverse-transcription quantitative PCR (RT-qPCR) for remodeling (*n* = 9/group) and inflammatory (*n* = 4/group) gene expression in whole-lung lobe lysates from double-transgenic (DTg) *Ccsp/ADAM33* or single-transgenic (STg) littermate control mice in which transgene expression was induced by doxycycline (Dox) feeding during pregnancy and for up to 28 days (28D on Dox) or 56 days (56D on Dox) after birth or Dox feeding for 28 days after birth followed by a cessation of Dox for 28 days (28D on + 28D off Dox): (**A**) *Acta2*, (**B**) *Col1a1*, (**C**) *Col3a1*, (**D**) *Fn1* (**E**) *Pecam1*, (**F**) *Ccl11*, (**G**) *Il5*, (**H**) *Il13*, (**I**) *Cxcl1*, and (**J**) *Muc5ac* (2-way ANOVA, Tukey’s multiple comparison test). Box plots show medians and 25th to 75th percentiles, and whiskers represent minimum and maximum values; all data points are shown. Results are from 3 independent experiments (**A–E**) and from 1 experiment (**F–J**). (**K–N**) Representative immunofluorescence staining for ACTA2/αSMA (green), PECAM1 (red), and nuclei (blue) in lungs from (**K**) STg littermate control or (**L**) DTg *Ccsp/Adam33* mice in which transgene expression was induced in utero and by Dox feeding postpartum for 28 days and (**N**) after 56 days. (**M**) DTg *Ccsp/Adam33* mice after ADAM33 transgene expression was induced in utero and by 28 days of Dox feeding postpartum and then 28 days off Dox. Aw, airway; Ve, vessel. Scale bar: 100 μm. Results are representative of 2 independent experiments (**K–N**).

As a susceptibility gene close to the origin of asthma, ADAM33 is a potential therapeutic target. However, as airway remodeling is considered to be a relatively irreversible process, therapeutic intervention depends on demonstration that sADAM33-induced airway remodeling can be reversed after sADAM33 expression is blocked. This concept was tested using the DTg *Ccsp/ADAM33* transgenic mice in which sADAM33 expression can be regulated by provision, or removal, of Dox from the diet. In these experiments, sADAM33 expression was commenced in utero and continued for 28 days postpartum (PD28) to promote a robust remodeling response; this was followed by 28 days without Dox feed to assess reversibility of the remodeling response (Supplemental Figure 10A). At day 56, when the analyses were performed, ADAM33 expression in the lungs of DTg *Ccsp/ADAM33* mice was completely ablated as a result of removing the Dox diet (Supplemental Figure 10B). At the same time, all airway remodeling genes, *Acta2*, *Col1a1*, *Col3a1*, *Fn1*, and *Pecam1*, had returned to baseline levels in whole lungs of DTg *Ccsp/ADAM33* mice and were at levels similar to those in STg littermate controls (Figure 8, A–E). Inflammatory markers *Ccl11*, *Il5*, *Il13*, and *Cxcl1* and goblet cell marker *Muc5ac* were not affected by the presence or absence of sADAM33 expression (Figure 8, F–J). Immunofluorescence histochemistry applied to lungs from 28- and 56-day-old *Ccsp/ADAM33* mice that were maintained on Dox in utero and throughout the whole experiment confirmed marked induction of airway remodeling, involving increased airway and vascular smooth muscle at both time points (Figure 8, L and N) compared with control mice (Figure 8K). However the lungs of DTg *Ccsp/ADAM33* mice on Dox diet for 28 days and then without Dox for 28 days showed a reduction in airway remodeling, with decreased staining for airway and vascular smooth muscle (Figure 8M), similar to the STg littermate control mice (Figure 8K).

## Discussion

In this report, we have defined the function of ADAM33 as a local tissue susceptibility gene for asthma and have uncovered a substantial interaction between sADAM33-mediated airway remodeling and sensitivity to allergen exposure, leading to allergic inflammation and BHR in early life. Ectopic expression of human sADAM33 in adult or fetal murine airways caused airway remodeling, which was completely reversible if expression of human sADAM33 was switched off. Almost identical structural changes (remodeling) were observed in human fetal lung in the presence of exogenous, enzymatically active sADAM33 but not the mutated, inactive enzyme. While on its own airway remodeling did not trigger inflammation or BHR in murine lungs, the remodeled lungs of mice expressing sADAM33 in utero were more susceptible to low amounts of HDM allergen exposure, resulting in augmented BHR and eosinophilia in early postnatal life. Since sADAM33 enhances the effects of low amounts of allergen to produce asthma-like features, sADAM33-induced airway remodeling could explain the difference in susceptibility of asthmatic individuals to environmental allergens, compared with atopic nonasthmatic subjects. This may be because sADAM33-induced airway remodeling results in increased numbers of fibroblast and smooth muscle cells that can produce mediators such as CCL11 (36, 37), which amplifies inflammation. Since sADAM33 is also proangiogenic (27), it may facilitate ingress of eosinophils or other inflammatory cells into the tissue in response to these chemoattractants.

The importance of ADAM33 for allergic airway responses was further highlighted using *Adam33^–/–^* mice in which HDM-induced airway inflammation, remodeling, and BHR were all markedly reduced in the absence of ADAM33. These data indicate an obligatory role for ADAM33 in the pathobiology of allergic airways disease via a mechanism involving loss of its membrane anchor,which produces a dysregulated protein (i.e., a gain of function). Consistent with this, we found that sADAM33-Pro-MP protein and enzymatic activity was increased in human asthma and that levels were not affected by corticosteroid treatment.

**Figure 9:**
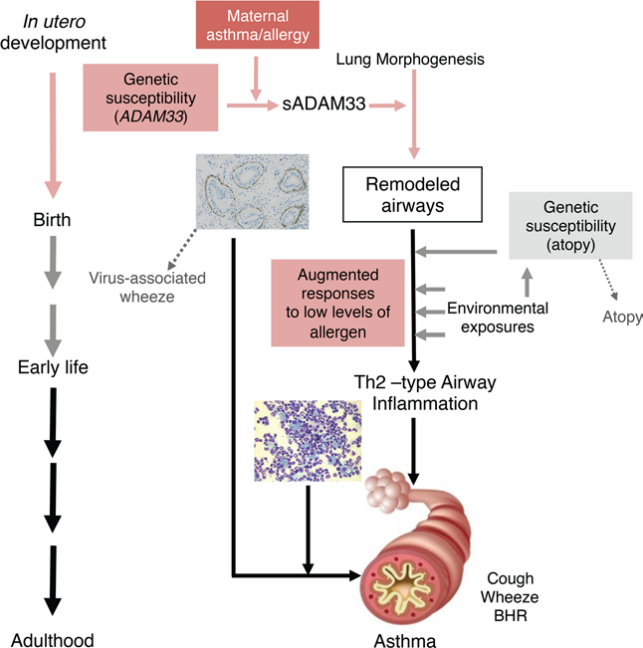
Schematic representation of the contribution of soluble ADAM33 (sADAM33) as a local tissue susceptibility gene in asthma pathobiology.

The ability of sADAM33 to promote airway remodeling is consistent with the association of *ADAM33* polymorphism with BHR (14) and reduced lung function (18–20). Furthermore, our observation that levels of sADAM33 in human asthma are unaffected by corticosteroids would help explain the inability of corticosteroid treatment to affect lung function in young children (8) or the natural history of the disease (7). Present findings challenge the classical paradigm of allergic asthma, in which Th2-driven airway inflammation has primacy over airway remodeling to result in the typical asthmatic phenotype (2, 4–6). Both inflammation and remodeling seem to be of importance and mutually cooperative at different stages of the life course. Since sADAM33 is induced in utero by maternal allergy (26), this may explain why airway remodeling (9) and bronchial smooth muscle (10) are increased in the airways of young children who subsequently develop asthma. These remodeled airways may provide the “soil” that supports exaggerated responses to allergens in the airways of susceptible individuals, leading to the Th2-type inflammation and BHR that characterizes asthma. Such a paradigm might explain the association of ADAM33 polymorphism with progression of preschool wheeze into childhood asthma (38) (Figure 9).

Individual approaches used in the current study have their inherent limitations. While specific expression of human sADAM33 might have been preferred in pulmonary mesenchymal cells that are the main site of ADAM33 expression (14, 21), the advantage of lung epithelial expression was that sADAM33 was released into the airway lumen, as observed in human asthma. The relevance of the transgenic model was further confirmed using WT mice in which murine sADAM33 was observed in BALF after allergen challenge; it was further supported by data from *Adam33*-null mice demonstrating that ADAM33 plays an essential role in the development of inflammation and remodeling in this model. One unresolved question is how sADAM33 is released and transferred into the lumen of the airways in asthma. Future work should focus on the role of disease-associated SNPs, especially those in the transmembrane and cytoplasmic domains (14), which might result in increased susceptibility for release of sADAM33 and, as a consequence, in stimulation of airway remodeling.

Currently, most asthma treatments are directed against Th2 inflammation (3, 39), with little effect on airway remodeling. The rationale for targeting the MP of ADAM33 is strongly supported by our human and mouse data, which both provide strong evidence for a key role of sADAM33 as an initiator of remodeling, independent of inflammation, that is prevented by a mutant inactive MP and is reversible when sADAM33 expression is arrested.

Although targeting MPs for diseases such as cancer has been historically problematic due to off-target effects (40), this problem may be overcome by development of specific small-molecule inhibitors of the sADAM33 MP based on the unique ADAM33 crystal structure (41, 42). Alternatively, the development of monoclonal antibodies, antisense nucleic acids, or specific microRNAs (43) may enable development of highly specific sADAM33 therapeutic approaches. Any of these strategies would be amenable to proof-of-concept testing using the murine models described herein prior to translation into human studies. Development of any such agent that successfully inhibits sADAM33-MP would be anticipated to have potential, as disease-modifying asthma therapies close to the origin of asthma (11).

## Methods

### Human samples.

BALF was obtained from healthy and asthmatic donors (Supplemental Table 1) by fiberoptic bronchoscopy performed in accordance to the British Thoracic Society guidelines (44) and standard operating procedures of the NIHR Wellcome Trust Clinical Research Facility and NIHR Southampton Respiratory Biomedical Research Unit at the University Hospital Southampton NHS Foundation Trust. BAL was performed by instilling 6 × 20 ml aliquots of prewarmed normal saline into a subsegmental bronchus of the anterior segment of the right upper lobe followed by gentle suction. Prior to processing, recovered BALF was filtered (BD Falcon cell strainer, Marathon Laboratory Supplies) and then centrifuged at 1,300 *g* for 10 minutes at 4°C. In order to prepare samples for cytospin and supernatant storage, the cell pellet was resuspended in PBS following removal of the supernatant, which was stored at –80°C for later analysis. Cytocentrifuged (Cytospin 4 Cytocentrifuge, Thermo Shandon Ltd.) cells were stained with rapid Romanowsky stain (Raymond Lamb Ltd.) for differential cell counts of macrophages, neutrophils, eosinophils, lymphocytes, and epithelial cells. A total of 400 cells was counted on coded samples by an operator unaware of the participant’s clinical characterization.

Human embryonic tissue was collected, staged, and processed as described previously (45). Gestational age was between 8 and 10 weeks. Human embryonic lungs were dissected into 1- to 2-mm pieces and cultured in Transwells in Matrigel (BD Bioscience) in the presence of 60 ng/well of active ADAM33 Pro-MP or inactive mutant E346A ADAM33 Pro-MP for 12 days, replenishing media and ADAM33 protein every second day as previously described (27). The tissue was harvested and processed for immunohistochemistry at day 12.

### Mice.

The animals were maintained in a pathogen-free environment. Food and water were provided ad libitum in temperature-controlled rooms on a 14 -hour light/10-hour dark cycle. Animals of mixed sex were used for experiments.

A pTRES-hADAM33-SS-PRO-MP-3Flag plasmid (human ADAM33 NCBI reference sequence: NM_025220.3) was generated as demonstrated in Supplemental Figure 4, A–G. The plasmid was digested with the restriction endonucleases *AatII* and *SapI* to obtain a linearized construct (Supplemental Figure 4F). This was purified and used for microinjection into pronuclei from FVB/N mice in the Transgenic Animal and Genome Editing Core Facility at the Cincinnati Children’s Hospital Medical Center. DNA was extracted from tails or ear tips of weanlings and used for PCR-based genotyping in order to find *ADAM33*-expressing founder mice (Supplemental Figure 4H). Founder mice were bred with *Ccsp-rtTA* (line 2) (33) to generate DTg *Ccsp/ADAM33* and STg littermate control mice that were used for phenotyping.

Expression of ADAM33 was induced in 6- to 8-week-old DTg (*Ccsp/ADAM33*) mice by provision of Dox (Lab Diet, 5LOS W/625 ppm Dox; TestDiet) in the food ad libitum for 4 and 8 weeks until the point of sacrifice. STg litter controls were fed Dox for the same time period (Supplemental Figure 5A).

*Ccsp/ADAM33* pregnant dams were given Dox in their diet during pregnancy and weaning; Dox was also given to their offspring until 4 weeks after birth. DTg *Ccsp/ADAM33* and STg littermate control offspring were sacrificed at ED17.5, PD10, and PD28 for further analysis (Supplemental Figure 5D).

For studies of the reversibility of airway remodeling, *Ccsp/ADAM33* pregnant dams were given Dox in their diet during pregnancy and weaning and their offspring were given Dox until PD28 and PD56. One group was given Dox for 28 days and then was fed without Dox for another 28 days. DTg *Ccsp/ADAM33* and STg littermate control offspring were sacrificed PD28 and PD56 for further analysis (Supplemental Figure 10A).

For the ADAM33 knockout experiments, sperm were obtained from the Mouse Biology Program at the University of California, Davis (a gift from Dean Sheppard and Chun Chen, Lung Biology Center, Department of Medicine, University of California) (35). This was used for in vitro fertilization to reconstitute *Adam33*^–/–^ mice in the Transgenic Animal and Genome Editing Core facility at Cincinnati Children’s Hospital Medical Center. Sperm from homozygote *Adam33*^–/–^ 129/SVJae mice were added to oocyte-cumulus complexes from superovulated FVB/N female mice for in vitro fertilization. Mixed background *Adam33^+/–^* mice were bred in order to generate a mix of *Adam33*^–/–^, *Adam33*^+/-^, and WT mice, which were genotyped as previously reported (35). Six- to eight-week-old offspring of mixed sex were used for the HDM extract challenge experiments according to the protocol described below (Supplemental Figure 3). The number of animals used for analysis was based on 3–5 animals per treatment group, and each experiment was repeated up to 3 times. This was based on power calculations (http://www.stat.ubc.ca) for invasive airway resistance measurements from a pilot experiment of *Adam33*^–/–^ and WT mice challenged with HDM. Assuming an 80% power, a 5% significance level, a 2-sided test, a common SD of 1.0–1.5, and that a difference in the mean airway resistance of 2.2–3.3 cm H_2_O/s/ml between DTg and control mice is likely to be of scientific interest, we estimated that sample sizes of about 2 to 8 mice would be required when testing concentrations of methacholine at 0, 25, 50, and 100 mg/ml.

Genotyping was performed before litters were weaned and split by gender. At the same time, animals were tagged with a metal clip or ear punches and given each a unique number. Animals with the appropriate genotypes were randomly included in the different experimental groups. The investigators were blinded during further analysis of the animals and processed samples using only the unique identification number.

### Genotyping PCR.

Primers used for PCR amplification of the conditional *Ccsp/ADAM33* mice were as follows: *ADAM33-PRO-MP* forward 5′-CAGCTTCTCAGGACTCTGGACATTC-3′ and reverse 5′-CGGGATCACTACTTGTCATCGTC-3′. About 100 ng of genomic DNA was PCR amplified using the following conditions: *Ccsp* — 94°C for 5 minutes, 30 cycles of 94°C for 30 seconds, 57°C for 30 seconds, and 72°C for 30 seconds, 72°C for 7 minutes, and hold at 4°C; *ADAM33-PRO-MP* — 94°C for 5 minutes, 30 cycles of 94°C for 30 seconds, 55°C for 30 seconds, and 72°C for 30 seconds, 72°C for 7 minutes, and hold at 4°C. We analyzed the PCR products on 1.5% agarose gel with ethidium bromide to examine the presence of the approximately 440-bp *Ccsp* and approximately 636-bp *ADAM33-PRO-MP* allele band. For genotyping of the *Adam33* mutant alleles, primers and conditions were used that had been described previously (35).

### IL-13 and HDM allergen challenges.

Six- to eight-week-old DTg *Ccsp/ADAM33* and STg littermate control mice were challenged with 5 μg of recombinant murine IL-13 or saline by intratracheal installation, and lungs were harvested for further analysis after 24 hours (Supplemental Figure 8A). Six- to eight-week-old age- and gender-matched FVB/N, *Adam33*-null, heterozygote and WT mice and DTg *Ccsp/ADAM33* and STg littermate control mice were sensitized intraperitoneally with HDM extract (*Dermatophagoides pteronyssinus* extract protein 8.1 mg/vial, lot 218862; Greer) (10 μg/200 μl saline) on days 0 and 7. On days 14 and 19 the mice were challenged intratracheally with 25 μg HDM/100 μl saline to induce allergic airway inflammation or with saline alone as control (Supplemental Figure 3). In addition, a dose response experiment was performed with lower concentrations of HDM (6.25, 12.5, and 25 μg/100 μl saline) on days 14 and 19 in WT mice, and the lowest dose was used for DTg *Ccsp/ADAM33* and STg littermate control mice that had been on Dox starting in utero (Supplemental Figure 9A). At day 21 after first sensitization, lung function was assessed (see below) and mice were harvested for BALF and lung tissue for further analysis (Supplemental Figure 3).

### Western blotting.

Human or murine BALF (from HDM-challenged mice) samples were concentrated and diluted using 2× or 6× Laemmli sample buffer before loading equal protein concentrations on to the gels. Samples were run on 10% Tris/glycine gels and transferred onto PVDF membranes. Where murine BALF samples were not concentrated, equal volumes of BALF were subjected to electrophoresis and transferred onto PVDF membranes. The transferred protein was assessed by Ponceau staining to ensure similar protein loading prior to blocking and Western blotting. Membranes were probed with polyclonal rabbit antibodies against the MP (ab39191, Abcam; 1:5,000) or the Pro (ab39190, Abcam; 1:5,000) domains of ADAM33 (Supplemental Figure 1) or a polyclonal goat antibody against the ectodomain of mouse ADAM33 (AF2434, R&D Systems; 1:1,000) as described previously (26, 28). Secondary antibodies were rabbit TrueBlot anti-rabbit IgG HRP antibody (18-8816, eBioscience; 1: 5,000) or rabbit anti-goat IgG HRP antibody (Calbiochem, Merck; 1:10,000). The blots were visualized using enhanced chemiluminescence (ECL+; GE Healthcare) with ImageQuant LS4000 or Amersham Imager 600 (GE Healthcare Life Sciences, Little Chalfont, UK). Semiquantitative analysis was performed by densitometry. Quantity One Analysis software (Bio-Rad) or ImageJ (National Institutes of Health, USA) was used to determine the intensity of the bands by measuring the integrated density as a product of area and mean gray value or by measuring relative density as result of the area of the lane profile plot (http://rsb.info.nih.gov/ij/docs/menus/analyze.html#gels). All human and mouse BALF samples were run on the same gel with positive control samples in form of Cos-7 cell lysates transfected with human ADAM33-MP-Pro and purified human recombinant ADAM33-MP-Pro protein or Cos-7 cell lysates transfected with full-length mouse ADAM33 (Mouse cDNA clone MR217277-20; OriGene) on each gel.

### Specificity testing of the human ADAM33 antibodies.

Human recombinant ADAM33-Pro-MP (produced in-house) and ADAM8-polyhistidine-tag (HIS) and ADAM12-polyhistidine-tag (HIS) (1031-AD-020 and 4416-AD-020; both from R&D Systems, BioTechne) were applied in 2-μl volumes containing 100, 75, 50, 25, or 0 ng of each protein onto pencil marked grids on PVDF membranes and air dried. After blocking, the membranes were incubated overnight with the polyclonal rabbit antibodies against the MP or Pro domains of ADAM33 (ab39191 and ab39190, Abcam; 1:5,000) or with mouse anti-HIS antibody (372900, GE Healthcare). After washing, bound antibody was detected using secondary antibodies: donkey anti-rabbit IgG HRP (NA9340V, GE Healthcare) or polyclonal rabbit anti-mouse HRP (P0260, Dako) and visualized using enhanced chemiluminescence (ECL+; GE Healthcare) with an Amersham Imager 600 (GE Healthcare Life Sciences) (Supplemental Figure 2).

### FRET peptide cleavage assay.

A FRET peptide cleavage assay was performed using a peptide that was selective for ADAM33. Analyses were performed using a StepOnePlus (Applied Biosystems, Life Technologies) or a Bio-Rad CFX96 (Bio-Rad) qPCR machine with a FAM (6-carboxyfluorescein) filter that measured fluorescence output every minute for 60 minutes. To assess enzymatic activity in BALF, each reaction was incubated with 7 μl of neat BALF at 37°C with 4.4 μM (0.5 μl of 88.7 μM) FRET peptide [DABCYL-YRVAFQKLAE(FAM)K-NH_2_] (41) (Severn Biotech) and 10 μM (0.5 μl of 200 μM) ZnCl_2_ in 2 μl of 5× assay buffer (100 mM HEPES, pH 7.0, 2.5 M NaCl, 50 mM CaCl_2_, and 1 mg/ml bovine serum albumin) in a total reaction volume of 10 μl. Enzymatic activity was determined by plotting the relative fluorescence units (RFU) against time after the background had been subtracted. The rate of the reaction (RFU/min) was determined from the line of best fit in the linear phase of the assay. Controls were recombinant soluble active ADAM33-PRO-MP and mutant ADAM33-PRO-MP (E356A) as previously published (27).

### qRT-PCR.

Lung tissue was either snap frozen or stored in RNAlater (Life Technologies) before homogenization and RNA extraction using Trizol Reagent (Invitrogen, Life Technologies). Genomic DNA contamination was removed by digestion with DNase (Life Technologies). First-strand cDNA was generated by reverse transcription using the RT-Standard cDNA synthesis kit (PrimerDesign). qPCR was performed using a CFX96 qPCR machine (Bio-Rad) for 40 cycles at 95°C for 5 seconds and 60°C for 20 seconds (fast protocol) or 40 cycles at 95°C for 15 seconds and 60°C for 60 seconds (standard protocol) followed by a melt curve analysis for SYBR green–based assays. All samples were run in duplicate. PCR product amplification was detected using PCR or SYBR green mastermix (PrimerDesign) or TaqMan Gene Expression Master Mix (Life Technologies) and primer sets without or with TaqMan/Perfect probes: *ADAM33-MP* forward 5′-CCTGGAACTGTACATTGTGGCA-3′, reverse 5′-GTCCACGTAGTTGGCGACTTC-3′, and FAM-probe 5′-CCACACCCTGTTCTTGACTCGGCAT-3′; *Acta2* forward 5′-TGAAGAGGAAGACAGCACAGC-3′, reverse 5′-GGAGCATCATCACCAGCGAA-3′, and FAM-probe 5′-CAGAGCCCAGAGCCATTGTCGCAC-3′; *Col1a1* forward 5′-TCGTGGCTTCTCTGGTCTC-3′, reverse 5′-CCGTTGAGTCCGTCTTTGC-3′, and Perfect probe 5′-CAGGGTCCTCCTGGTTCTCCTGGTTCTCGACCCTG-3′; *Col3a1* forward 5′-ATATGCCCACAGCCTTCTAC-3′, reverse 5′-CAGGAATGCCAGGAGGAC-3′, and Perfect probe 5′-CTGCTCCTGTGCTTCCTGATGGCCAGCAG-3′; *Fn1* forward 5′-AAGAGGACGTTGCAGAGCTA-3′ and reverse 5′-AGACACTGGAGACACTGACTAA-3′; and *Pecam1* forward 5′-TCCAACAGAGCCAGCAGTAT-3′ and reverse 5′-GCAGAGAGCAATACAGAGGAA-3′ (PrimerDesign) and *Ccl11* Mm00441238_m1; *Il5* Mm00439646_m1; *Il13* Mm00434204_m1; *Cxcl1* Mm04207460_m1; *Muc5ac* Mm01276718_m1; and *Gapdh* Mm99999915_g1 (Life Technologies). Relative mRNA expression was quantified using 2^–ΔΔCt^ method (46).

### Luminex multiplex analysis.

Undiluted murine BALF samples were analyzed using a mouse Magnetic Luminex Screening assay containing a premixed multi-analyte kit for murine Ccl11 and IL-5 according to manufacturer instructions (R&D Systems) on a Luminex 200 (Luminex).

### Inflammatory cell counts.

Murine BALF samples were collected by washing the lungs 3 times with 800–1,000 μl sterile PBS (PBS). The total volume of the combined fluids was measured and centrifuged at 300 *g* for 5 minutes. The BALF supernatants were frozen for analysis of sADAM33. Red blood cells were lysed from the cell pellets, which were subsequently resuspended in 300 μl PBS. Cells were counted, and 100,000 cells were loaded into a cytospin funnel and centrifuged at 300 *g* for 5 minutes on to a glass slide. Slides were air dried, and the cells were stained using a Diff-Quick stain (Sigma-Aldrich) followed by fixation and H&E staining. The different inflammatory cell types were counted to a total of 300 cells and expressed as the differential cell count in cells/ml BALF.

### Assessment of lung function.

Mice were anesthetized with 100 μl of triple anesthetic containing a 4:1:1 mixture of ketamine, acepromazine, and xylazine by intraperitoneal injection. A FlexiVent machine (Scireq) was used to assess lung function in the form of airway resistance (R) after aerosolized methacholine challenge to provide a measure of BHR, as described by the manufacturer’s instructions. Airway resistance was measured by forced oscillation technique, with increasing values indicating bronchoconstriction of the lungs. BHR measurements were obtained from individual animals using increasing stepwise concentrations of 0, 25, 50 and 100 mg/ml methacholine in saline for the *Ccsp/ADAM33* and HDM extract–challenged mice. On completion of lung function assessment, mice were sacrificed and BALF and lung tissue were collected for further analyses.

### Histochemistry and immunofluorescence-histochemistry.

Lungs were inflation-fixed with 4% paraformaldehyde or 10% neutral buffered formalin at 25 cm of water pressure for 5 minutes and then fixed overnight before embedding in paraffin wax. Five-μm-thick serial sections were cut for histological staining using H&E or immunofluorescence-histochemistry using standard protocols. We used a FITC-conjugated mouse monoclonal antibody against ACTA2 (αSMA), 1:250 (F3777; Sigma-Aldrich); a primary rabbit anti-human ADAM33-PRO, 1:1,000 (ab39190, Abcam), with a secondary Alexa Fluor 594 goat anti-rabbit IgG, 1:200 (Invitrogen); and a primary rat antibody against murine CD31 (PECAM1), 1:75 (DIA 310; Dianova, Stratech Scientific Ltd.), with a secondary Alexa Fluor 647 goat anti-rat IgG, 1:500 (112-605-003; Jackson Immune Research, Stratech Scientific Ltd). Stained slides were mounted with a cover slip in ProLong Anti-Fade Gold (Life Technologies), which contained DAPI as nuclear counterstain. Human embryonic lungs were acetone-fixed and embedded in glycol methacrylate resin. Consecutive 2-μm sections were cut and immunostained with a mouse monoclonal antibody against ACTA2 (αSMA), 1:40,000 (A2547, Sigma-Aldrich) using standard protocols (21, 47). Lung sections were examined by light microscopy using a Zeiss Axioplan 2 (Carl Zeiss Microscopy) or a Leica AF6000 LX (Leica Microsystems) microscope equipped with AxioVision software (Zeiss) or LAS AF software (Leica Microsystems).

### Statistics.

Normal distribution of the numeric data was evaluated, and the appropriate parametric or nonparametric statistical tests were used. Statistical significance was assessed by using the 2-tailed Student’s *t* test (parametric, unpaired data) with Welch’s correction if SDs were not equal or Mann Whitney test (nonparametric, unpaired data) for comparisons between 2 groups. For comparison of 3 or more groups a 1-way ANOVA with Tukey’s multiple comparison test (parametric data) or Kruskal-Wallis test with Dunn’s test for correction for multiple comparisons (nonparametric data) was used. For comparison of 2 or more groups with 2 independent variables, a 2-way ANOVA with Tukey’s multiple comparison test was used (Prism 6, version 6.0e, Graphpad). *P* values of less than or equal to 0.05 were considered significant. Data are presented as mean ± SD or median with 25% and 75% interquartiles, with all data points shown. Whiskers represent minimum and maximum values.

### Study approval.

Collection of bronchoscopy samples was performed after approval from the Southampton and South West Hampshire Joint Local Research Ethics Committee and after informed consent was obtained from the donors. Human embryonic lungs were harvested according to the Polkinghorne Committee guidelines after approval from the Southampton and South West Hampshire Joint Local Research Ethics Committee and obtaining informed consent from the donor. For all mouse experiments, the 3R (Replacement, Reduction, and Refinement) principles were followed and experiments were conducted according to the Animal Research: Reporting of In Vivo Experiments (ARRIVE) guidelines (48), the guidelines for the care and use of animals approved by the Institutional Animal Care and Use Committee at the Cincinnati Children’s Hospital Research Foundation (Cincinnati, Ohio, USA), and the local Southampton University ethical committee under project and personal licenses from the Home Office, United Kingdom.

## Author contributions

ERD helped design and carried out most experiments, analyzed data, and contributed to writing the manuscript. JFCK performed murine lung function and RNA expression studies. PHH provided human adult samples and clinical data and edited the manuscript. DIW provided human fetal lungs and edited the manuscript. STH acted as advisor and edited the manuscript. DED contributed to experimental design, interpretation of the data, and cowrote the manuscript. JAW supervised research and design of the ADAM33 transgenic mouse, provided critical reagents and laboratory space, and edited the manuscript. HMH supervised the project, designed and performed experiments, analyzed data, and was the lead coauthor of the manuscript.

## Supplementary Material

Supplemental data
