# Supplementary material for: Soluble ADAM33 initiates airway remodeling to promote susceptibility for allergic asthma in early life
Source: JCI Insight. 2016 Jul 21;1(11):e87632. doi: 10.1172/jci.insight.87632 (PMC4968941; doi:10.1172/jci.insight.87632)
Supplement: Supplemental data [file jciinsight-1-87632-s001.pdf]

Soluble ADAM33 initiates airway remodeling to promote susceptibility for allergic asthma in early life

Elizabeth R. Davies, Joanne F.C. Kelly, Peter H. Howarth, David I Wilson, Stephen T. Holgate, Donna E. Davies, Jeffrey A. Whitsett, Hans Michael Haitchi

Supplemental data

Supplemental Table 1. Subject characteristics of bronchoalveolar lavage fluid (BALF) groups.

	Healthy	Asthma
Subjects	(n=5)	(n=10)
Age, yrs	19 (19-21)	42 (21-69)*
Male/Female	4/1	3/7
Atopy	0	6
FEV1% predicted	96 (83-101)	70 (30-101)*
SABA	N/A	10
ICS	N/A	7
LABA	N/A	7
OCS	N/A	2
Other meds	N/A	3
Eosinophilic only	1	2§
Neutrophilic only	0	3§
Eosin.+Neutr.	0	4§
Neither	4	1

* Unpaired Student's t-test with Welch's correction ($P < 0.05$)
§ Number of patients with eosinophilic (≥1%), neutrophilic (≥5%) or both inflammation in bronchoalveolar lavage fluid (BALF)
Inhaled short-acting ß2-agonists (SABA), Inhaled corticosteroids (ICS), Inhaled long-acting ß2-agonists (LABA), Oral steroids (OCS), Other medications (Other meds), not applicable (N/A)

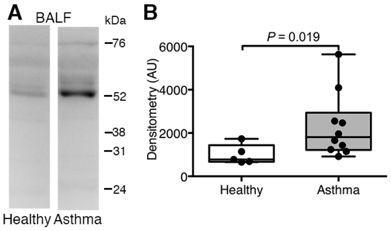

Supplemental Figure 1: Increased soluble ADAM33 (sADAM33) Pro-Metalloprotease in bronchoalveolar lavage fluid (BALF) in human asthma. (**A**) Western blotting of BALF proteins from healthy (n=5) and asthmatic (n=10) subjects using an antibody recognizing the Pro domain of human ADAM33; representative blots are shown. (**B**) Combined ADAM33 immunoreactive bands (at ~52 - 76kDa) were analyzed by densitometry in arbitrary units (AU) (Mann Whitney test). Box plots show medians and 25th to 75th percentiles, and whiskers min to max/all points. Full unedited Western blots are available in the Supplemental Material.

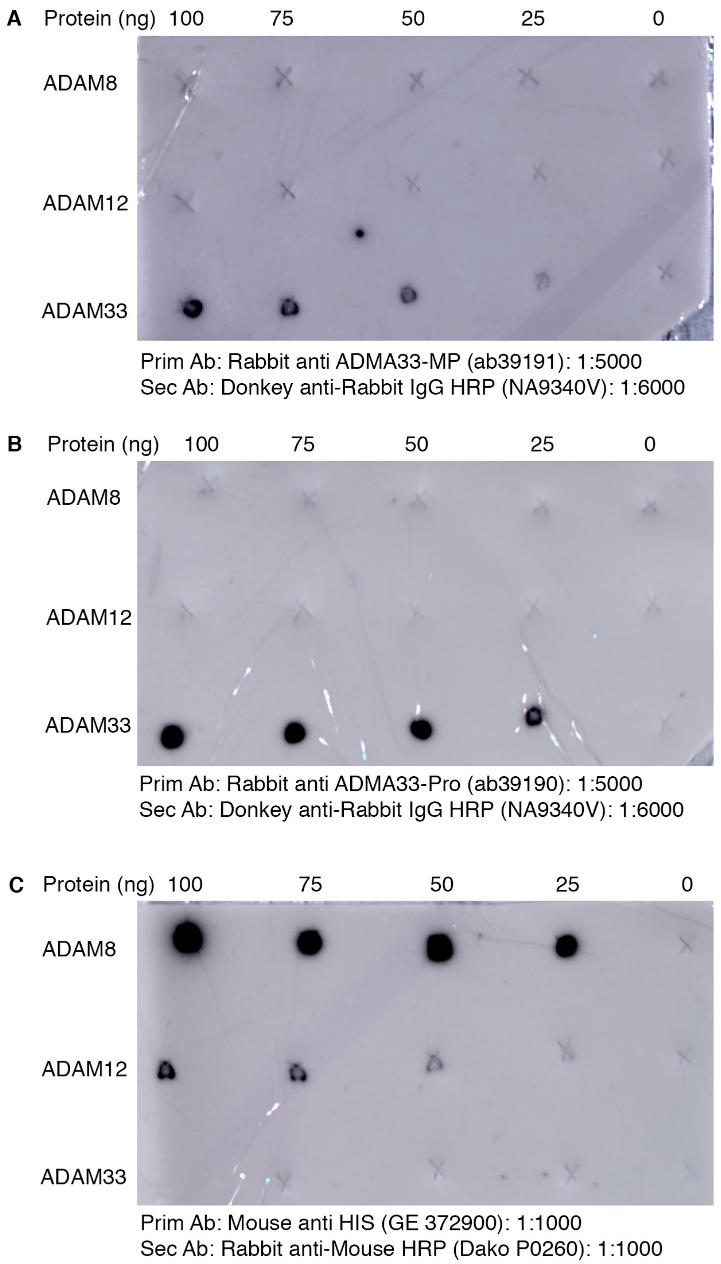

Supplemental Figure 2: ADAM33 metalloprotease (MP) and pro domain (Pro) antibodies do not crossreact with ADAM8 or ADAM12. (**A,B,C**) Dot blots of 100, 75, 50, 25 and 0 ng recombinant ADAM8, ADAM12 (both with a polyhistidine (HIS) tag) and ADAM33 blotted with antibodies against (**A**) ADAM33-MP, (**B**) ADAM-Pro and (**C**) HIS tag.

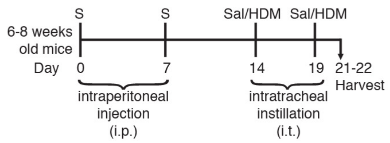

Supplemental Figure 3: Mouse model of house dust mite (HDM)-induced allergic airway inflammation

Protocol for intra-peritoneal (i.p.) sensitization (S) of 6-8 week old mice at day 0 and day 7 and intra-tracheal (i.t.) challenges with Saline (Sal) or HDM extract in saline (HDM) on day 14 and 19. Harvesting (Harvest) of mouse bronchoalveolar lavage fluid (BALF) and lung tissue (Harvest), on day 21-22 after first sensitization.

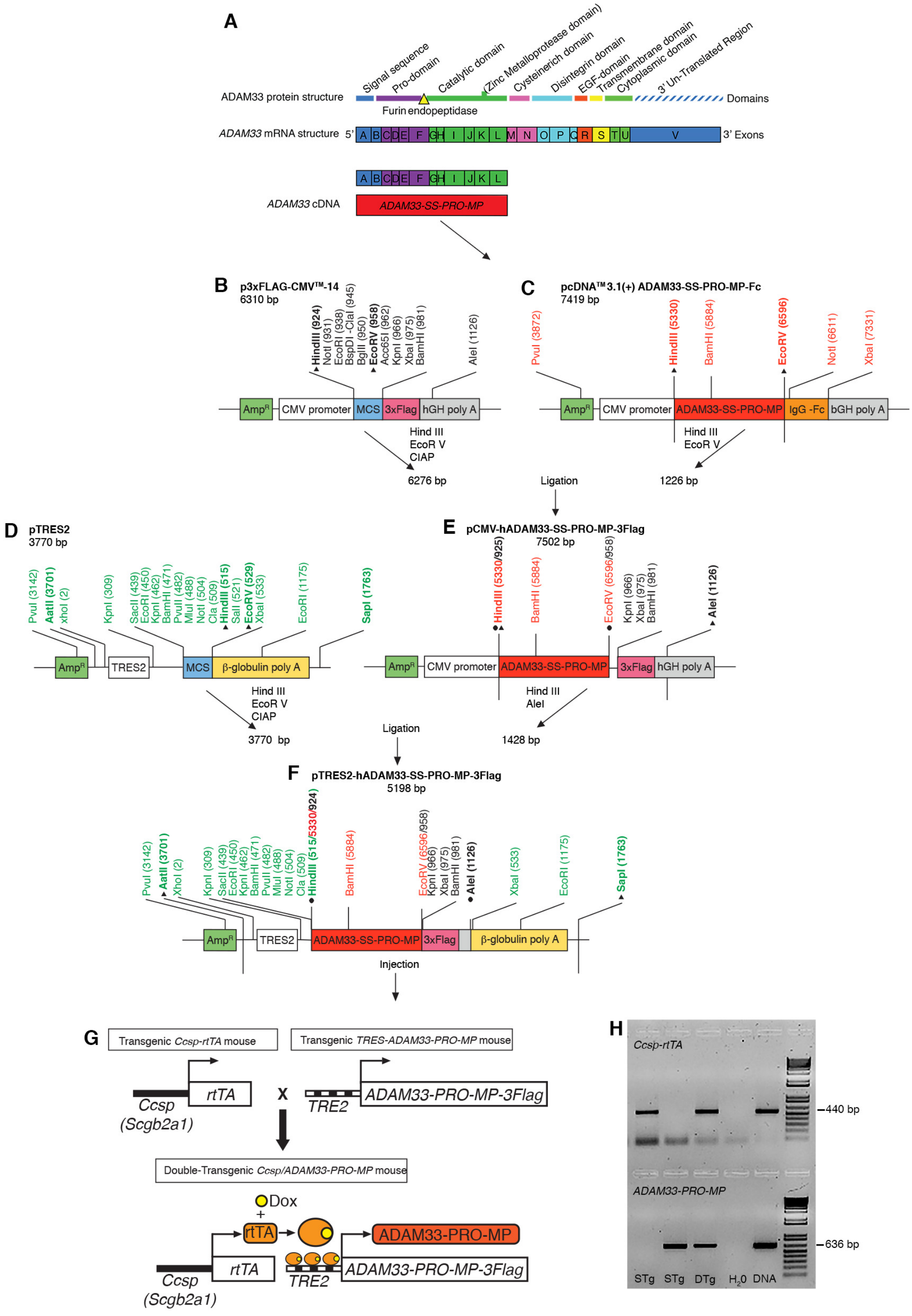

A
ADAM33 protein structure
Signal sequence
Pro-domain
Catalytic domain
(Zinc Metalloprotease domain)
Cysteinerich domain
Disintegrin domain
EGF-domain
Transmembrane domain
Cytoplasmic domain
3' Un-Translated Region
Domains
Furin endopeptidase
ADAM33 mRNA structure 5'
3' Exons
ADAM33 cDNA
ADAM33-SS-PRO-MP
B p3xFLAG-CMV™-14
6310 bp
AmpR
CMV promoter
MCS
3xFlag
hGH poly A
Hind III
EcoR V
CIAP
6276 bp
C pcDNA™3.1(+) ADAM33-SS-PRO-MP-Fc
7419 bp
IgG -Fc
bGH poly A
1226 bp
Ligation
D pTRES2
3770 bp
TRES2
β-globulin poly A
3770 bp
E pCMV-hADAM33-SS-PRO-MP-3Flag
7502 bp
AleI
1428 bp
F pTRES2-hADAM33-SS-PRO-MP-3Flag
5198 bp
Injection
G
Transgenic Ccsp-rtTA mouse
Transgenic TRES-ADAM33-PRO-MP mouse
rtTA
Ccsp (Scgb2a1)
X
TRE2
ADAM33-PRO-MP-3Flag
Double-Transgenic Ccsp/ADAM33-PRO-MP mouse
Dox
ADAM33-PRO-MP
H
Ccsp-rtTA
440 bp
ADAM33-PRO-MP
636 bp
STg STg DTg H2O DNA

Supplemental Figure 4: Generation of the human sADAM33 double transgenic (*Ccsp/ADAM33*) mouse model.
(**A**) Diagram of the full length ADAM33 protein domain structure, the corresponding mRNA exonic structure and the cDNA used for cloning sADAM33 which contains the signal sequence (SS), pro-domain (PRO) and metalloprotease (MP) domain. (**B-F**) Plasmid constructs and steps for generation of the linearized TRES-hADAM33-SS-PRO-MP-3Flag DNA construct for microinjection into pro-nuclei from FVB/N mice. **(G)** *Ccsp-rtTA* mice line 2 were crossed with founder mice containing the tetracycline response element 2-*ADAM33-PRO-MP* (*TRES-ADAM33-PRO-MP*) to generate Doxycycline (Dox)-inducible double transgenic mice expressing human soluble ADAM33 (*Ccsp/ADAM33*). (**H**) Agarose gel electrophoresis of PCR products from *Ccsp-rtTA* (440 base pairs (bp)) or *ADAM33-PRO-MP* (636 bp) transgenes. Results from DNA of single (STg; *Ccsp-rtTA* or *ADAM33-PRO-MP*) or double transgenic (DTg; *Ccsp/ADAM33*) mice, and negative (H2O) and positive control DNAs are shown.

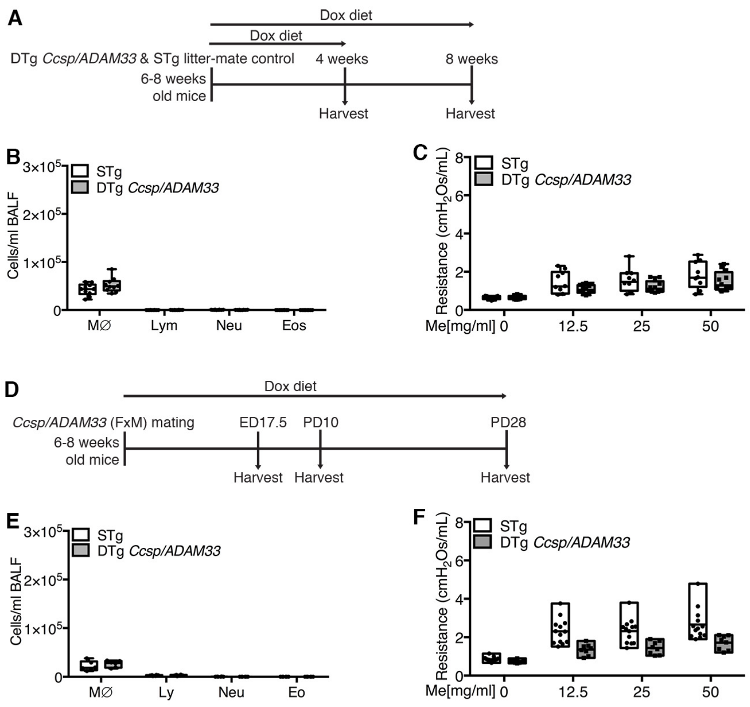

Supplemental Figure 5: Expression of sADAM33 does not induce airway inflammation or airway resistance.

(**A**) Six to eight week old double transgenic (DTg) *Ccsp/ADAM33* (n=8-12) and single transgenic (STg) litter-mate control (n=8-10) mice were fed on Dox diet for 8 weeks. (**B**) shows differential inflammatory cell counts for macrophages (M∅), lymphocytes (Ly), neutrophils (Neu) and eosinophils (Eo) in bronchoalveolar lavage fluid (BALF) from adult DTg *Ccsp/ADAM33* or STg control mice (n=8 per group) for 8 weeks on Dox diet; (**C**) shows airway resistance in response to increasing concentrations of methacholine (Me) in phosphate buffered saline (PBS) in adult DTg Ccsp/ADAM33 (n=12) and litter STg control (n=10) mice. (**D**) DTg *Ccsp/ADAM33* (n=6-13) and STg litter-mate control (n=6-8) were fed on Dox from in utero until 4 weeks post partum to induce transgene expression. (**E**) shows differential inflammatory cell counts in BALF from young DTg *Ccsp/ADAM33* or STg control mice on Dox from in utero for 4 weeks (n=6 per group); (**F**) shows airway resistance in response to increasing concentrations of methacholine (Me) in phosphate buffered saline (PBS) in DTg *Ccsp/ADAM33* (n=13) and litter STg control (n=8) in 4 week old mice on Dox from in utero (two-way ANOVA, Tukey's multiple comparison test). Results are from 3 independent experiments (**B,C,E,F**).

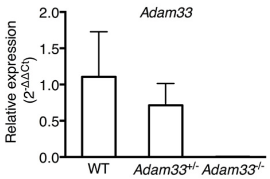

Supplemental Figure 6: *Adam33* mRNA expression in wild type, heterozygous and *Adam33*$^{-/-}$ mice.

Relative mRNA expression of *Adam33* in whole lung lysates from wild-type (WT), heterozygous (*Adam33*$^{+/-}$) and ADAM33 knock out (*Adam33*$^{-/-}$) mice (n=9/group) was determined by reverse transcription quantitative PCR (RT-qPCR) analysis.

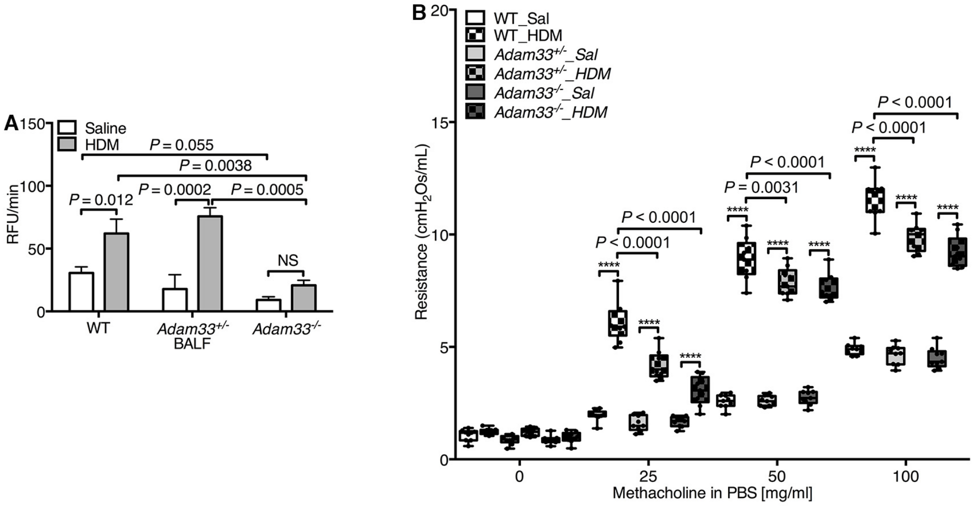

Supplemental Figure 7: Bronchoalveolar lavage fluid (BALF) enzymatic activity and airway resistance are decreased in *Adam33*$^{-/-}$ mice following HDM challenge.

Wild-type (WT), heterozygote (*Adam33*$^{+/-}$) and ADAM33 knock out (*Adam33*$^{-/-}$) mice were sensitized and challenged with saline or house dust mite extract HDM following the standard protocol. (**A**) shows ADAM33 enzymatic activity measured using an ADAM33-selective fluorescence resonance energy transfer (FRET) peptide cleavage assay using BALF from mice after HDM or saline challenge as indicated (n = 3/group) (two-way ANOVA, Tukey's multiple comparison test). Data are expressed as mean ± s.d. Results are from 1 experiment (**B**).

(**B**) shows airway resistance in response to increasing concentrations of methacholine in PBS measured using the forced oscillation technique in anesthetized mice after HDM or saline challenge as indicated (n=9 per group) (one-way ANOVA, Tukey's multiple comparison test). Data are expressed as medians, boxes 25th to 75th percentiles and whiskers min to max/all points. Results are from 3 independent experiments (**B**

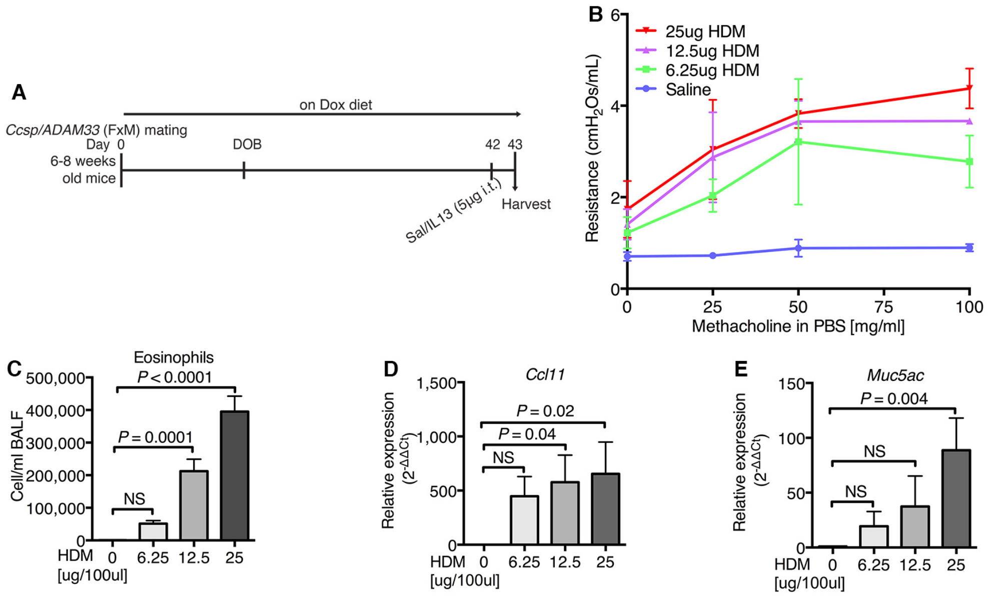

Supplemental Figure 8: IL13 challenge model and concentration dependence of house dust mite extract (HDM) on bronchial hyperresponsiveness (BHR) and eosinophilic inflammation.

(**A**) Ccsp/ADAM33 mice were mated and double transgenic (DTg) *Ccsp/ADAM33* and single transgenic (STg) litter-mate control offspring mice were on Doxycycline (Dox) diet from *in utero* until day 42 after birth to induce transgene expression; they were then challenged with saline (Sal) or 5 μg of murine IL13 by intra-tracheal (i.t.) installation and harvest of lung tissue after 24 hours (n=4 per group). (**B-E**) Wild-type mice were sensitized and challenged with saline or 6.25, 12.5 or 25 μg of HDM in saline (n=3 per group) following the standard protocol. (**B**) shows airway resistance in response to increasing concentrations of methacholine in PBS measured using the forced oscillation technique in anesthetized mice after HDM or saline challenge as indicated; (**C**) shows differential inflammatory cell count for eosinophils (Eo) in bronchoalveolar lavage fluid (BALF) from mice after HDM or saline challenge as indicated (one-way ANOVA, Tukey's multiple comparison test). (**D,E**) shows mRNA expression

measured by reverse transcription quantitative PCR (RT-qPCR)using whole lung lysates from mice after HDM or saline challenge as indicated: (**D**) *Ccl11/Eotaxin* and (**E**) *Muc5ac* (one-way ANOVA, Tukey's multiple comparison test). Data are expressed as mean ± s.d. Results are from 1 experiment (**B-E**).

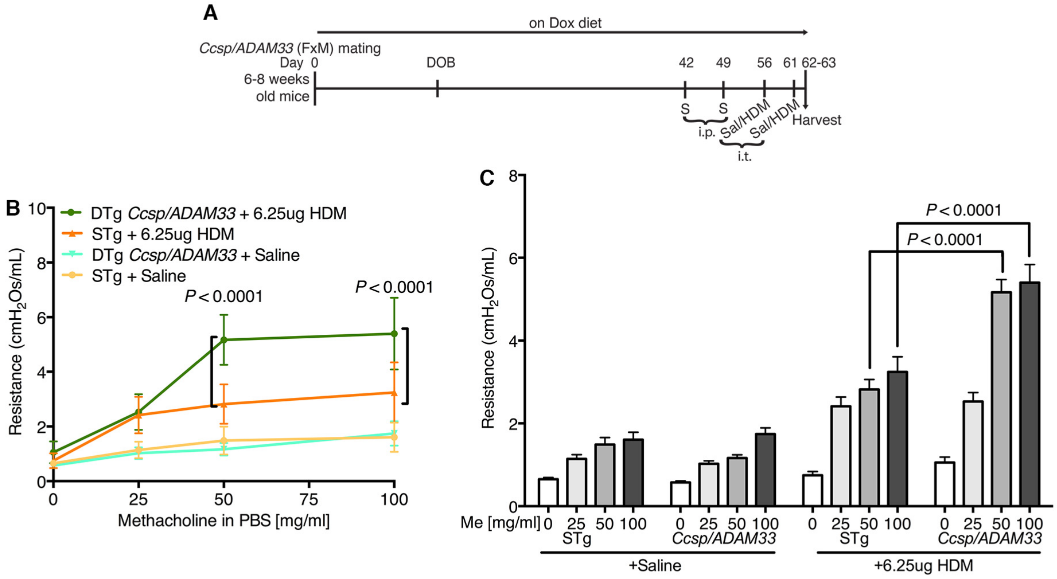

Supplemental Figure 9: Increased airway responsiveness after exposure to a low concentration of house dust mite extract (HDM) allergen in human soluble ADAM33 (sADAM33) expressing transgenic mice.
(**A**) Double transgenic (DTg) *Ccsp/ADAM33* (n=9/group) and single transgenic (STg) litter-mate control (n=9/group) mice were fed on Doxycycline (Dox) diet for 6 weeks to induce transgene expression; they were then sensitized and challenged with 6.25 µg of HDM or saline. (**B,C**) show airway resistance in response to increasing concentrations of methacholine (Me) in PBS measured using the forced oscillation technique in anesthetized mice after HDM or saline challenge as indicated (two-way ANOVA, Tukey's multiple comparison test). Data are expressed as mean ± s.d. Results are from 3 independent experiments (**B** and **C**).

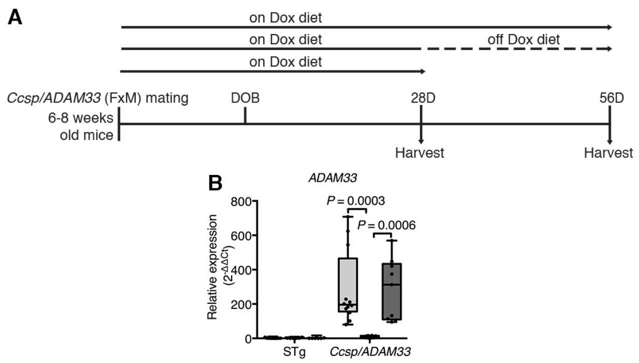

Supplemental Figure 10: ADAM33 expression is arrested after 28 days on and 28 days off Doxycycline (Dox).

(**A**) Double transgenic (DTg) *Ccsp/ADAM33* and single transgenic (STg) litter-mate control were fed on Dox from in utero until 28 and 56 days post partum to induce transgene expression. In one group Dox was stopped for 28 days to arrest transgene expression. (**B**) shows reverse transcription quantitative PCR (RT-qPCR) for *ADAM33* mRNA expression in whole lungs from DTg *Ccsp/ADAM33* or STg control mice on Dox from in utero for 28 (light gray boxes) and 56 days (dark gray boxes) and 28 days on followed by 28days off Dox (white boxes) (n=9 per group) (two-way ANOVA, Tukey's multiple comparison test). Results are from 2 independent experiments (**B**).

Full unedited blots for Figure 1A (top) & Supplementary Figure 1A (bottom):

Western blots: BALF from Healthy and Asthmatic Subjects

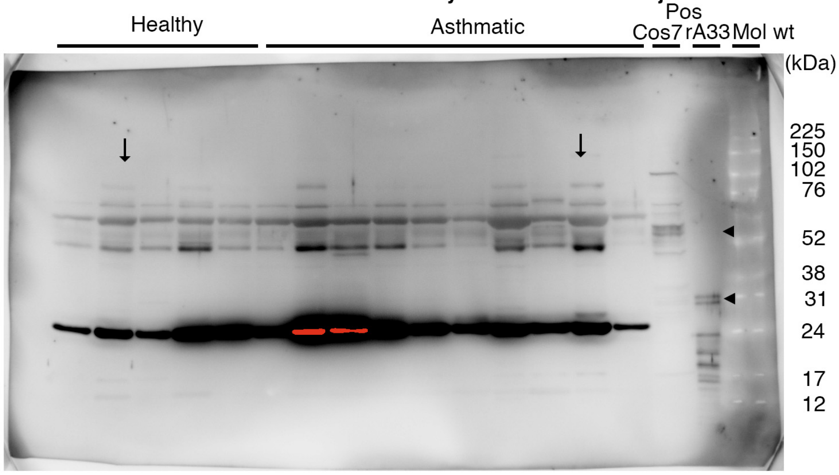

Western blot: using Rabbit anti-ADAM33-MP antibody (ab39191)
Arrows indicate representative lanes used in Figure 1A; Healthy controls (n=5) and Asthmatic Patients (n=10); Pos = Positive controls: Cos7-cells lysate tranfected with human ADAM33-MP-Pro (unproccessed double band ~55kDa (arrow head)); rA33: purified recominant ADAM33-MP-Pro protein (processed MP domain, double band ~30kDa (arrow head)); Mol wt = Molecular weight; Equal protein concentrations were loaded into each gel well.

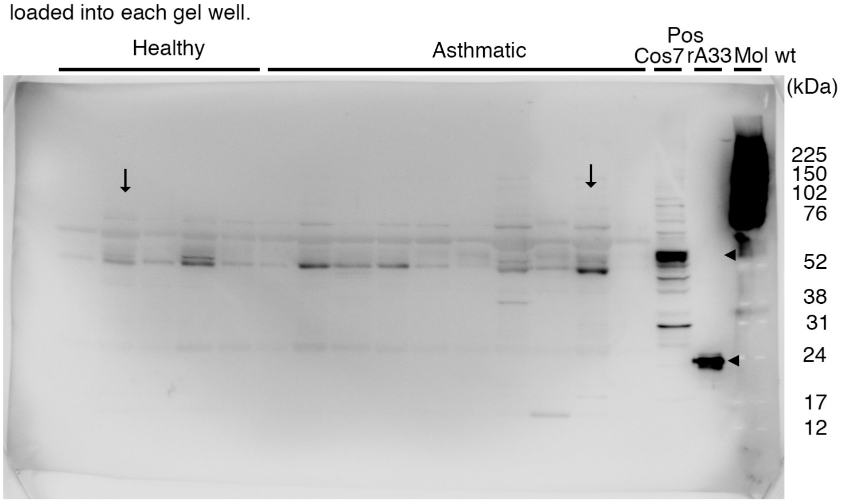

Western blot: using Rabbit anti-ADAM33-Pro antibody (ab39190):
Arrows indicate representative lanes used in Supplemenal Figure 1A; Healthy controls (n=5) and Asthmatic Patients (n=10); Pos = Positive controls: Cos7-cells lysate tranfected with human ADAM33-MP-Pro (unproccessed double band ~55kDa (arrow head)); rA33: purified recominant ADAM33-MP-Pro protein (processed Pro domain, band ~24kDa (arrow head)); Mol wt = Molecular weight; Equal protein concentrations were loaded into each gel well.

Full unedited blot for Figure 1D:

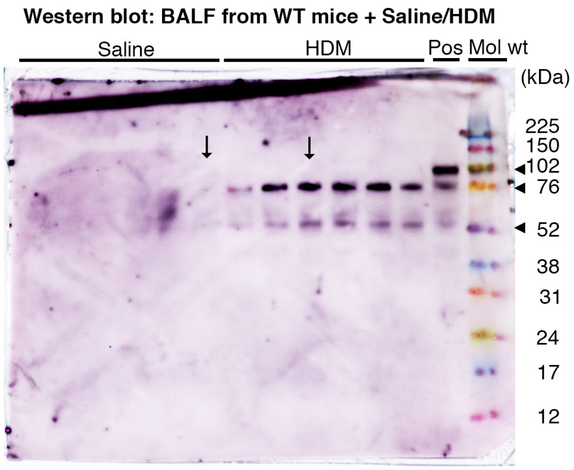

Arrows indicate representative lanes used in Figure 1D; Saline (n=6); HDM = House Dust Mite extract (n=6); Pos = Positive control: lysate from Cos-7 cells tranfected with full length mouse ADAM33 (OriGene Mouse cDNA clone MR217277-20); Mol wt = Molecular weight. The 100kDa band (arrwo head) = processed full length ADAM33 and the bands at 52 and 76kDa (arrow heads) the cleaved ectodomain.

Full unedited Ponceau stain and blot for Figure 2D:

Ponceau stain: *Ccsp/ADAM33* **lung lysates**

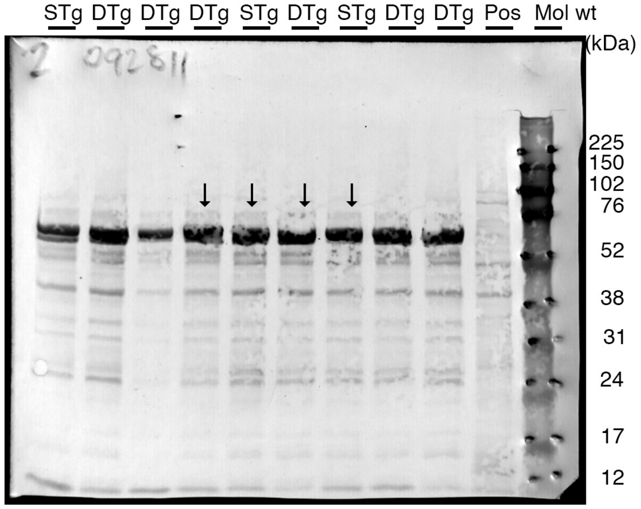

Western blot: *Ccsp/ADAM33* **lung lysates**

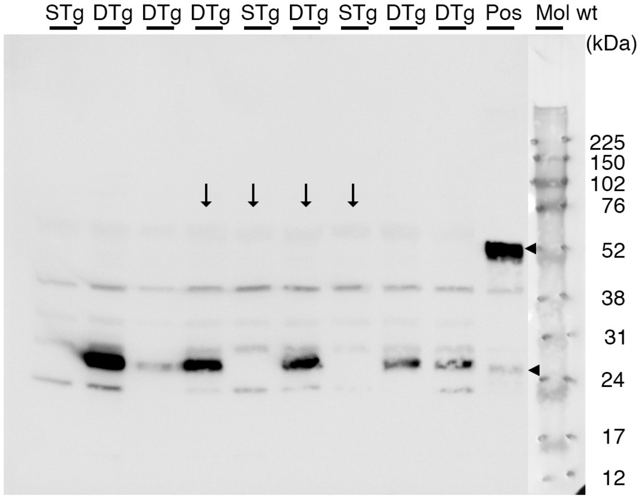

Arrows indicate representative lanes used in Figure 2D; *Ccsp/ADAM33* mouse lung lysates: DTg = double transgenic, STg = single trangenic; Pos = Positive control:lysate from Cos-7 cells tranfected with human ADAM33-MP-Pro. The band at 52kDa (arrow head) is unprocessed MP-Pro and the band approx. 25kDa (arrow head) the processed Pro domain); Mol wt = Molecular weight.
